# Induction of durable remission by dual immunotherapy in SHIV-infected ART-suppressed macaques

**DOI:** 10.1126/science.adf7966

**Published:** 2024-02-29

**Authors:** So-Yon Lim, Jina Lee, Christa E. Osuna, Pratik Vikhe, Dane R. Schalk, Elsa Chen, Emily Fray, Mithra Kumar, Nancy Schultz-Darken, Eva Rakasz, Saverio Capuano, Ruby A Ladd, Hwi Min Gil, David T. Evans, Emily K. Jeng, Michael Seaman, Malcolm Martin, Christiaan Van Dorp, Alan S. Perelson, Hing C. Wong, Janet D. Siliciano, Robert Siliciano, Jeffrey T. Safrit, Douglas F. Nixon, Patrick Soon-Shiong, Michel Nussenzweig, James B. Whitney

**Affiliations:** 1 Center for Virology and Vaccine Research, Beth Israel Deaconess Medical Center, Harvard Medical School, Boston, MA 02215, USA; 2 Wisconsin National Primate Research Center, University of Wisconsin-Madison, Madison, WI 53715, USA; 3 Department of Medicine, Johns Hopkins University School of Medicine, Baltimore, MD 21205, USA; 4 Altor Biosciences, Miramar, FL 33027, USA; 5 Laboratory of Molecular Microbiology, National Institute of Allergy and Infectious Diseases, National Institutes of Health, Bethesda, MD 20892, USA; 6 Los Alamos National Laboratory, Los Alamos, NM 87545, USA; 7 Santa Fe Institute, Santa Fe, NM 87501, USA; 8 Howard Hughes Medical Institute, Johns Hopkins University School of Medicine, Baltimore, MD 21205, USA; 9 ImmunityBio, Culver City, CA 90232, USA; 10 Division of Infectious Diseases, Department of Medicine, Weill Cornell Medicine, New York, NY 10065, USA; 11 Laboratory of Molecular Immunology, The Rockefeller University, New York, NY 10065, USA; 12 Howard Hughes Medical Institute, The Rockefeller University, New York, NY 10065, USA; 13 Department of Biology, Boston College, Chestnut Hill, MA 02467, USA

## Abstract

The eradication of the viral reservoir represents the major obstacle to the development of a clinical cure for established HIV-1 infection. Here, we demonstrate that the administration of Anktiva (N-803) and broadly neutralizing antibodies (bNAbs) resulted in sustained viral control after discontinuation of antiretroviral therapy (ART) in SHIV-AD8–infected, ART-suppressed rhesus macaques. N-803+bNAbs treatment induced immune activation and transient viremia, but only limited reductions in the SHIV reservoir. Upon ART discontinuation, viral rebound occurred in all animals, which was followed by durable control in approximately 70% of all N-803+bNAb-treated macaques. Viral control correlated with the reprogramming of CD8^+^ T cells by N-803+bNAb synergy. Thus, complete eradication of the replication-competent viral reservoir is likely not a prerequisite for the induction of sustained remission after discontinuation of ART.

The latent SIV/HIV-1 reservoir is established in CD4^+^ T cells within the first few days after infection and cannot be eliminated by the host immune system or current antiretroviral drug regimens (1–5). This archive of replication-competent virus is the source of viral rebound in most persons living with HIV-1 (PLWH) who discontinue ART and is thought to represent a critical hurdle for HIV-1 eradication strategies (6).

N-803 (Anktiva) is a soluble IL-15 superagonist complex that has been shown to markedly potentiate the mobilization and expansion of natural killer (NK) and CD8^+^ T cell populations. N-803 is being evaluated in preclinical and clinical trials for the treatment of various oncology indications and, most recently, for HIV-1 (7–10). Potent HIV-1 envelope–specific broadly neutralizing antibodies (bNAbs) have been shown to reduce viremia in untreated, chronically SHIV-infected rhesus macaques (RMs) and in PLWH (11–15). Therapeutic bNAbs have also been shown to potentiate CD8^+^ T cell responses in both SHIV-infected RMs and PLWH (11–14, 16, 17). Studies have shown that bNAb treatment under ART suppression can stimulate the control of viremia for up to 4 months (18). Here, we demonstrate that the administration of N-803 combined with bNAbs can induce long-term viral control following ART discontinuation in SHIV-infected, ART-suppressed RMs. The primary mechanism of viral control is associated with the augmentation of CD8^+^ T effector function induced by N-803+bNAbs.

A total of 36 Indian-origin RMs were intrarectally (IR) infected with SHIV-AD8 for two separate studies (Fig. 1A). All RMs were screened prior to study entry to exclude select MHC class I alleles. Once infection had progressed to the early chronic stage at weeks 7 to 8 postinfection, all RMs were administered antiretroviral therapy (ART) as a subcutaneous daily injection of tenofovir disoproxil fumarate, emtricitabine, and dolutegravir (19). Plasma SHIV RNA was monitored throughout the study and declined rapidly on ART. Once suppressed, viremia remained undetectable in all RMs (Fig. 1, B and D).

## Combined N-803 and 10-1074 therapy in adult rhesus macaques

**Fig. 1.**
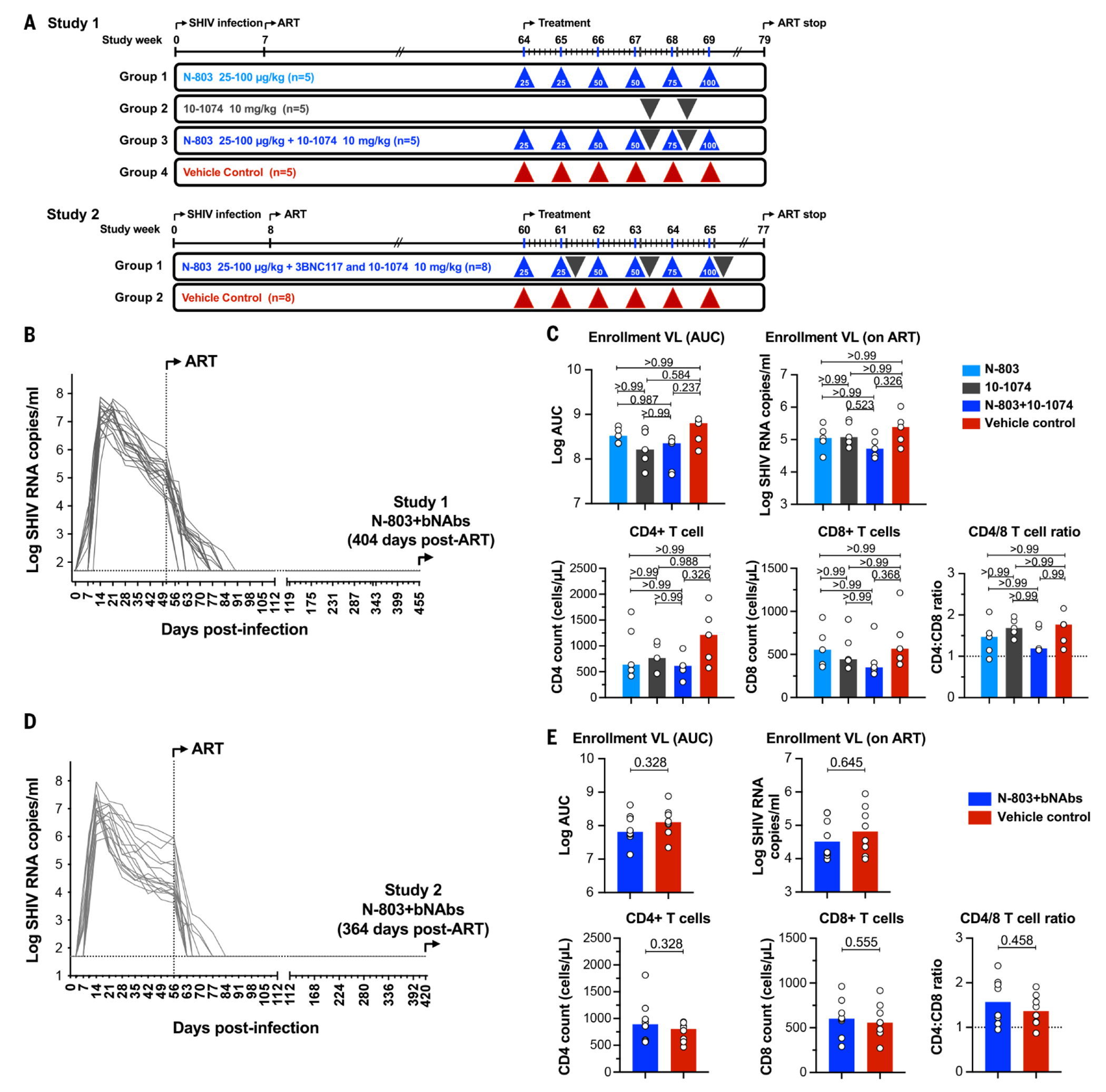
Study schema of N-803 and bNAb dosing. Diagrammatic overview of each study is shown. In Study 1, Groups 1, 2, and 3 (n=5 per group) received N-803 alone, 10-1074 alone, or N-803+10-1074, respectively. Group 4 (n=5) received vehicle alone as a control. N-803 was dose-escalated in Groups 1 and 3 with weekly SC administration. Two doses of 10-1074 at 10 mg per kilogram of body weight were administered IV in groups 2 and 3, 1 week apart. In Study 2, the treatment group (n=8) received N-803 in combination with 10-1074+3BNC117. N-803 was dosed at the same concentration and frequency as used in Study 1. Three doses of 10-1074+3BNC117 were administered EOW starting 24 hours after the second N-803 dose. Control group (n=8) received formulation vehicle only. (**A**) The schedule for SHIV-AD8 infection, ART initiation, therapeutic treatment and discontinuation of ART are shown as vertical lines (in weeks). (**B**) In Study 1, viral RNA was monitored in longitudinally from the day of SHIV-AD8 infection to the initiation of N-803+bNAb therapy. Twenty SHIV-infected ART-suppressed animals were then distributed into three experimental groups and one control group (n=5 per group). (**C**) Animals were distributed among groups by balancing virologic and immunologic metrics. Plots show the median values The comparison between groups was determined using a Kruskal–Wallis *H* test. *P*-values are adjusted for multiple comparisons. (**D**). In Study 2, viral RNA was monitored in RMs from the day of SHIV-AD8 infection to the initiation of N-803 and bNAb therapy. Sixteen SHIV-infected ART-suppressed RMs were then assigned to experimental and control groups (n=8 per group). (**E**) Animals were distributed among groups by balancing viral and immunologic parameters. Plots show the median with all values. Comparisons between groups were determined using a Mann–Whitney *U* test.

Study 1 used 20 MHC-defined, SHIV-AD8-infected RMs (Fig. 1A). These RMs were ART-suppressed for 404 days prior to initiating N-803+bNAb treatment (Fig. 1B). RMs were distributed into four arms (n=5 per arm) based on balancing virologic and immunologic entry criteria between each arm (Fig. 1C). Prior to intervention, no significant differences in these criteria were observed between arms. Group 1 received N-803 alone, Group 2 received 10-1074 alone, and Group 3 received a combination of N-803+10-1074. Control animals in Group 4 received formulation vehicle only. N-803 was delivered by six escalating SC injections, dosed weekly. Twenty-four hours after the fourth N-803 dose, we initiated two successive 10-1074 administrations spaced 1 week apart in Groups 2 and 3 (Fig. 1A).

After each N-803 administration, alone or in combination with 10-1074, we observed a significant pharmacodynamic (PD) response. We observed potent activation of T and NK cells. Peak cell activation, as measured by CD69, was observed approximately 48 hours after each N-803 dose. CD8^+^ T effector memory (TEM) cells expressed high levels of CD69 after each N-803 dose. By contrast, naïve and central memory (TCM) CD8^+^ T cell subsets expressed no CD69 following administration of N-803 (fig. S1A). CD4^+^ TEM cells and NK cells, but not naive or TCM CD4^+^ T cells, also highly expressed CD69 in response to N-803 administration (fig. S1, B and C).

**Fig. 2.**
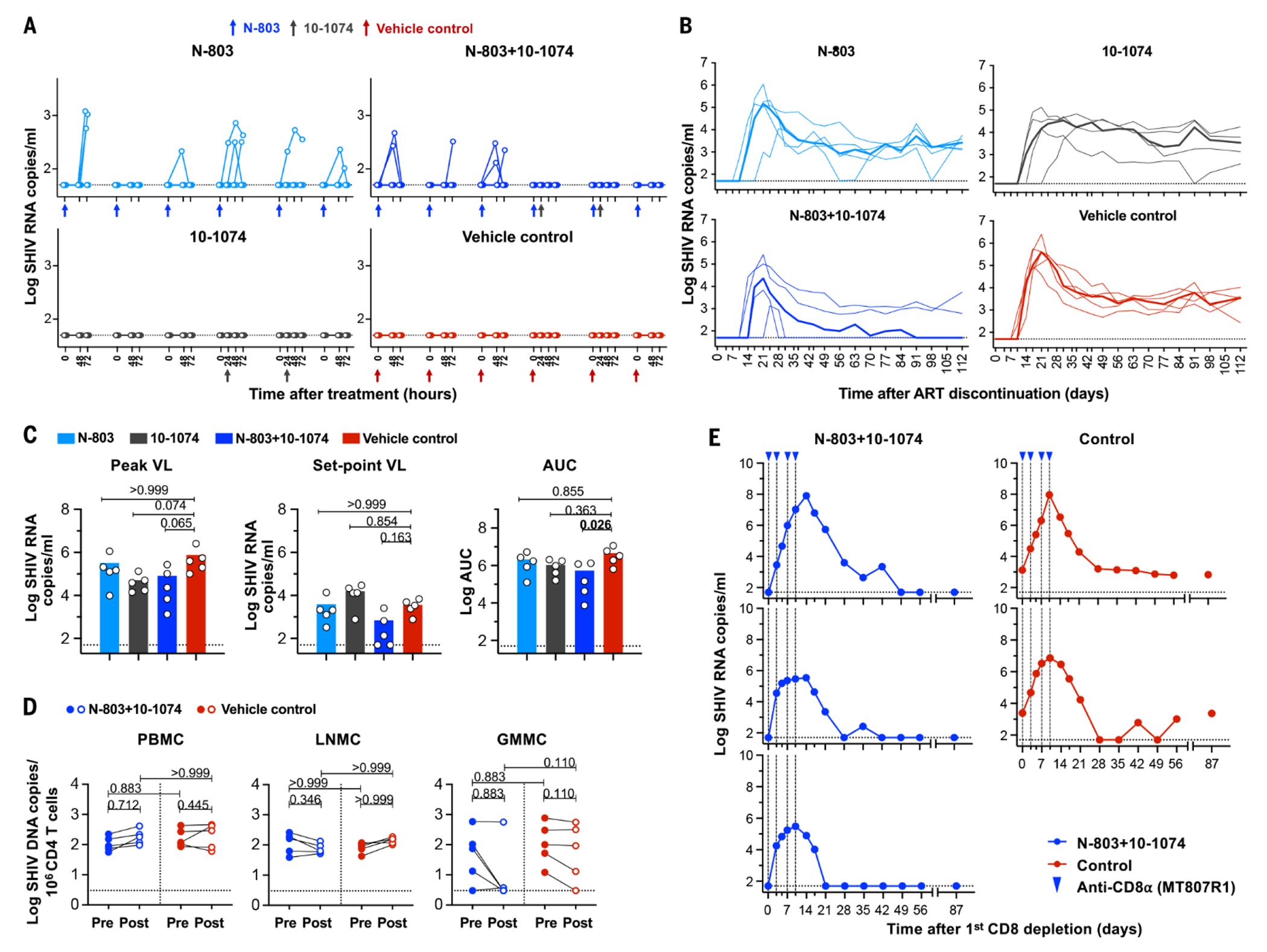
Viral and immune modulation following N-803+bNAb treatment. (**A**) Transient plasma viremia induced by N-803 in SHIV-infected monkeys on ART. SHIV RNA copies were monitored in RMs at baseline, 48, and 72 hours following each of six weekly N-803 doses in all treatment and control groups. Arrows indicate the timing of both N-803 and bNAb dosing. ART was discontinued after complete washout of 10-1074 in each group. (**B**) Plasma log viral RNA copies were assessed on days 0, 3, 7, 10, and 14 and then weekly until 112 days following ART discontinuation. Median values are indicated as bold lines. **(C)** Viral RNA levels at the time of peak, set point and total viral burden calculated as AUC are shown. Statistical comparisons between groups were assessed using a Mann–Whitney *U* test. (**D**) Change in integrated SHIV DNA in CD4^+^ T cells following N-803+10-1074 treatment: integrated SHIV DNA is expressed as log10 copies per 10^6^ CD4^+^ T cells at pre- and post-treatment timepoints (before ART discontinuation). Differences in integrated viral DNA at the time of pre- and post-treatment between groups was compared using a Kruskal–Wallis *H* test and changes in viral DNA in each group post treatment was compared to pre-treatment using a Wilcoxon matched-pairs signed-rank test. *P*-values were adjusted for multiple comparison by Dunn’s test. (**E**) CD8^+^ lymphocytes were depleted in vivo using the MT807R19 antibody. Doses were delivered to RMs that received N-803+10-1074 or two controls RMs, as indicated by blue triangles. Viral RNA was monitored longitudinally after anti-CD8α depletion.

We next evaluated the impact of N-803 on plasma viremia in ART-suppressed RMs. In Study 1, we detected transient plasma viremia in all RMs that were administered N-803 (n=10) (Fig. 2A). Episodes of transient viremia were intermittent, often reaching their peak between 48 and 72 hours after each N-803 administration, then returning to baseline by the next N-803 dose. The proportion of RMs exhibiting transient viremia showed dose-to-dose variations (fig. S2). Transient viremia was not detected in RMs after 10-1074 infusion or in control RMs (Fig. 2A).

There were minimal PD perturbations to T or NK cells after 10-1074 dosing (fig. S1, A to C). A faster pharmacokinetic (PK) clearance of 10-1074 was observed in RMs that also received N-803 administration, although this did not reach statistical significance (fig. S3). One RM displayed an anti-drug antibody (ADA) response after the second infusion of 10-1074.

Once washout of 10-1074 antibody was confirmed in all RMs, ART was then discontinued (fig. S3). Rebound viremia occurred in all RMs (Fig. 2B) and we observed no significant differences in the time to viral rebound between groups (Kaplan–Meier log-rank test, *P*=0.096).

We next assessed viral peak, set point and the total viral burden for each group following ART discontinuation (Fig. 2C). Treated groups were not significantly different from controls with regard to peak viremia (*P*=0.059) or viral burden (AUC, *P*=0.069) but were significant for virologic set point (*P*=0.026) (Fig. 2B). This reduction in setpoint was particularly prominent in three of five RMs that received N-803+bNAb treatment (Fig. 2C) compared to controls. During rebound, all three animals displayed an abbreviated low-level acute viremia, followed by viral control. Thus, the post-treatment virologic control observed in these three RMs, once achieved, was durable for all 301 days of follow up monitoring.

## The correlates of viral control in Study 1

We next sought to determine if N-803+bNAb therapy had altered the size or composition of the SHIV reservoir, resulting in the viral control observed in the three N-803+bNAb-treated RMs. We found that integrated viral DNA was largely unchanged in peripheral blood mononuclear cells (PBMCs) and lymph node mononuclear cells (LNMCs) between pre- and post-treatment timepoints in treated and control groups (Fig. 2D). Integrated viral DNA in CD4^+^ T gastrointestinal mucosa mononuclear cells (GMMCs) declined to undetectable levels in several treated animals, although this variability may have also reflected some degree of sampling inconsistency in this anatomic tissue (Fig. 2D).

To determine if the control of recrudescent viremia manifest in the three N-803+bNAb-treated RMs was due to antiviral immunity, we performed an in vivo CD8^+^ lymphocyte depletion on day 301 after ART discontinuation. The three aviremic RMs and two viremic control RMs received four infusions of the anti-CD8α antibody MT807R1. After infusion, CD8^+^ cells were cleared from the periphery for at least 14 days (fig. S4). In the absence of CD8^+^ lymphocytes, aviremic and viremic control animals rebounded with a rapid spike in plasma viral RNA (Fig. 2E). As CD8^+^ T and NK cells recovered, viremia in the treated RMs returned to undetectable. The three treated RMs were monitored for an additional 87 days and all RMs remained aviremic (Fig. 2E). One control RM remained viremic, whereas a second control RM had oscillating viremia but by day 56 postdepletion remained continuously viremic until the end of monitoring.

N-803 and dual bNAb therapy in adult rhesus macaques.

In Study 2, we sought to confirm and extend our observations regarding the induction of viral remission by N-803+bNAb therapy. This study used 16 SHIV-AD8-infected RMs (Fig. 1A), ART was then initiated at 7-8 weeks after IR infection. All RMs were ART-suppressed for 364 days before N-803+bNAb administration (Fig. 1D). Prior to treatment, all RMs were distributed into one of two groups (n=8 per group) based the same criteria used in Study 1 (Fig. 1E). The treatment group in this study received the same N-803 regimen used in Study 1. However, the bNAb regimen was modified to include three doses of two different bNAbs. In group 1, both 10-1074 and 3BNC117 antibodies were coadministered (at 10 mg per kilogram of body weight each) every other week (EOW, 24 hours after N-803 administration). Control RMs (group 2) received formulation vehicle at the same frequency (Fig. 1A).

**Fig. 3.**
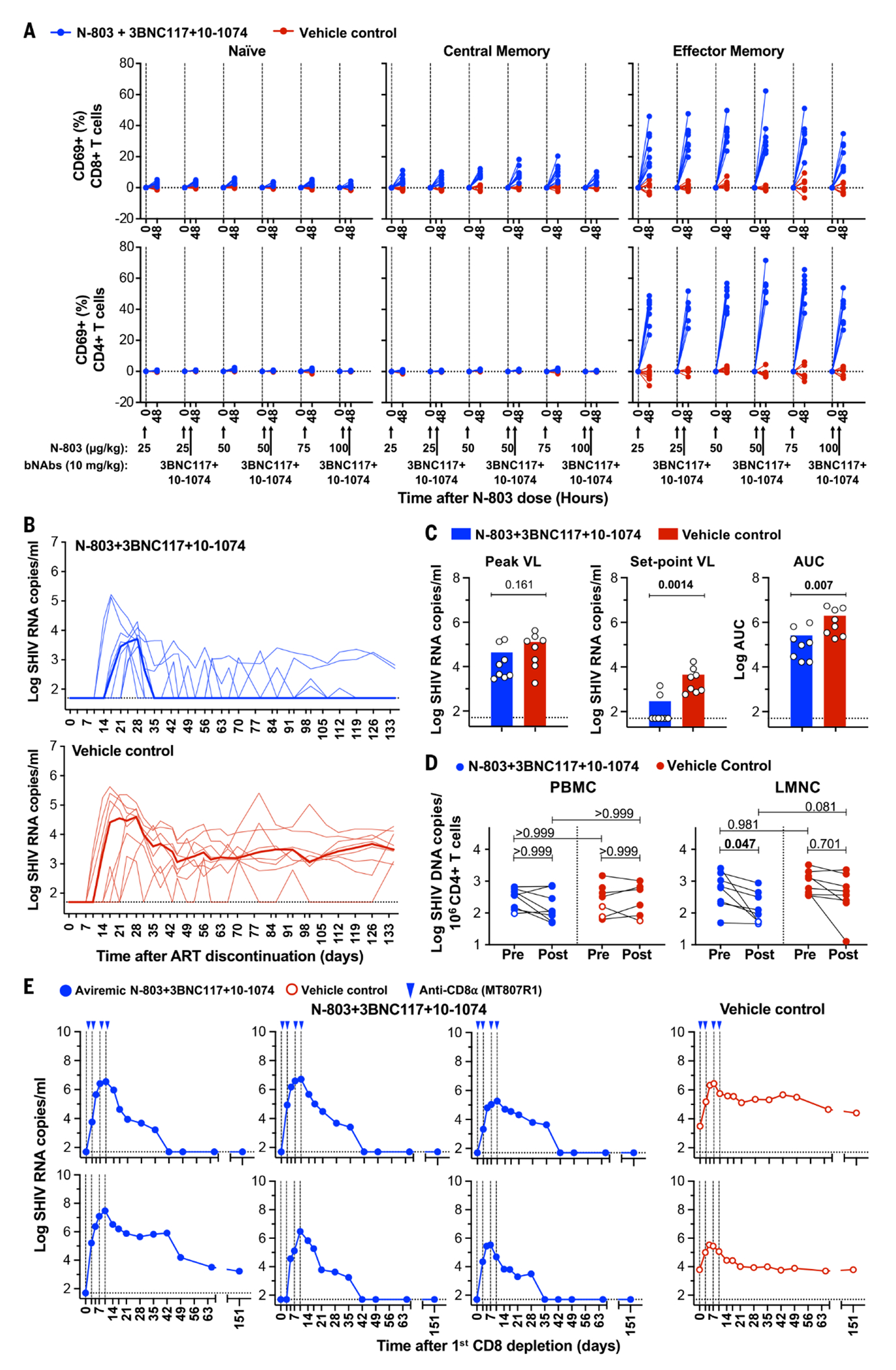
Viral and immune modulation following ART discontinuation and CD8^+^ lymphocyte depletion. The activation of T cells following N-803+10-1074 administration: Activation of naïve (CD95^−^CD28^+^), central memory (CD95^+^CD28^+^), and effector memory (CD95^+^CD28^−^) populations from CD8^+^ and CD4^+^ T cells was monitored by flow cytometry–based measurement of CD69. (**A**) CD69 expression within each subset was measured at the time of each dose (baseline) at 48 hours post each N-803 dose. Changes in CD69 expression are shown as the absolute difference in percent from the day of dose for all immune subsets. N-803 and 10-1074 dosing are indicated by black arrows on the *x*-axis. (**B**) Viral rebound following ART discontinuation after bNAb washout. Median values are indicated as bold lines. (**C**) Viral RNA levels at the time of peak, set point and AUC are shown. Statistical comparisons between groups were assessed using a Mann–Whitney *U* test. (**D**) Intact SHIV DNA in CD4^+^ T cells prior to and after treatment with N-803+bNAbs. Intact SHIV DNA by IPDA is expressed as log10 copies per sorted 10^6^ CD4^+^ T cells. The results are shown as the mean of intact SHIV genomes per 10^6^ CD4^+^ T cells calculated from three replicates, corrected for 2LTR circles and DNA shearing (filled symbols). Open symbols represent the limit of detection for samples in which no positive events were detected across three replicates, based on the number of cell equivalents assayed. Differences in intact viral DNA between groups at the time of pre- and post-treatment were compared using a Kruskal–Wallis *H* test and intact DNA at post-treatment were compared to pre-treatment using a Wilcoxon matched-pairs signed-rank test. *P*-values were adjusted for multiple comparison by Dunn’s test. (**E**) In vivo CD8^+^ lymphocyte depletion. CD8^+^ lymphocytes were depleted in vivo using monoclonal antibody MT807R1 in two control animals and all six treated remission RMs. The presence of CD8α^+^ lymphocytes in the blood was monitored by flow cytometry. Log viral RNA was assessed at the intervals indicated.

After N-803+bNAb administration, we again observed potent PD responses in T and NK cells. In CD8^+^ and CD4^+^ T cells, after each N-803 dose, CD69 expression was largely restricted to EM subsets (Fig. 3A). Increased CD25 expression was generally restricted to CM subsets (fig. S5A). NK cells reacted similar to those in Study 1 (fig. S5B). Both CD8^+^ and CD4^+^ TCM and TEM populations displayed rapid margination into tissues within 48 hours after N-803 doses. However, only CD8^+^ TCM and TEM subsets sustained margination across all six N-803 doses (fig. S5C). We also noted the increased expression of CXCR3 and CXCR5 on CD8^+^ T cell subsets following treatment in LN, consistent with increased trafficking to lymph node germinal centers (fig. S5, D and E). N-803+bNAbs administration also resulted in a significant net expansion of CD8^+^ TCM and TEM cells over time (TCM *P*<0.0001, EM *P*=0.0001). This net change was not observed in CD4^+^ T subsets, limiting concern over reservoir expansion by N-803 (fig. S5F).

In Study 2, we also detected transient viremia after N-803 dosing. Transient viral RNA was observed following the first and second N-803 dose but was not detected once bNAbs were administered. Control RMs were consistently aviremic (fig. S6).

We monitored bNAb PK throughout the study (fig. S7 and table S2) and prior to ART discontinuation and we confirmed that the washout of 3BNC117, and 10-1074 antibody had occurred (fig. S7). ART was then discontinued, and all RMs rebounded (Fig. 3B). The time to viral rebound was not significantly different between groups (Kaplan–Meier log-rank test P=0.131). In six of eight N803+bNAb-treated RMs, we noted rebound kinetics similar to that observed in the three aviremic treated-RMs from Study 1. These six RMs displayed a brief viral rebound and then a period of osillating low-level viremia followed by sustained viral control that was achieved by approximately day 80 after ART discontinuation. The median virologic set-point in N-803+bNAb-treated RMs was significantly reduced compared to controls (*P*=0.0014) as was the total viral burden (*P*=0.007) (Fig. 3C). This virologic remission was durable and persisted for all 210 days of follow-up monitoring.

## The correlates of viral control in Study 2

To determine the impact of our modified N-803+bNAb regimen on the viral reservoir, we performed an intact proviral DNA assay (IPDA) (Fig. 3D and fig. S8). SHIV DNA was isolated from purified CD4^+^T PBMCs, LNMCs or GMMCs at pre- and post-treatment time points from both groups. In PBMC, intact provirus was largely unchanged between pre- and post-treatment timepoints from both groups. However, we did detect a significant reduction of intact virus in CD4^+^ T LNMCs after treatment (median 0.35 log, *P*=0.047). In GMMCs, nucleic acid recovery was insufficient for IPDA analysis. Reservoir reductions also differed markedly among N-803+bnAb–treated RMs. The LMNC of the six treated aviremic RMs had a 0.39 median log reduction in intact SHIV DNA as compared to a 0.12 median log reduction in the two viremic treated RMs. In controls, no significant changes in intact viral DNA were noted (*P*=0.701) (Fig. 3D).

With limited reductions in the viral reservoir, we sought to confirm if the viral control manifest in the six N-803+bNAb-treated animals was immune-mediated. At 210 days after ART discontinuation, we depleted CD8^+^ lymphocytes in vivo in the six aviremic treated and two viremic control RMs, as described for Study 1. After infusion, CD8^+^ cells were depleted for 14-28 days (fig. S9) and all RMs rebounded with a rapid spike in plasma viral RNA. As CD8^+^ cells recovered, five of the six N-803+bNAb-treated RMs quickly suppressed viremia back to undetectable (Fig. 3E and fig. S9). The sixth RM controlled viremia approximately 79 days later. In total, we monitored animals for 245 days after CD8α^+^ depletion all treated RMs remained aviremic. Controls remained viremic during this period.

Reprogrammed CD8^+^ T cells appear to be one possible correlate of viral control. However, we could not discount a role for antibody-dependent cell-mediated cytotoxicity (ADCC) or autologous neutralizing antibodies contributing to viral suppression. We evaluated ADCC using plasma isolated from treated or control RMs from Study 2 at three timepoints: 24 hours after the third bNAb dose, at the time of ART discontinuation, and then 4 weeks after ART discontinuation. Plasma isolated from the eight treated animals showed 50% SHIV-AD8 specific ADCC activity in the presence of 10-1074+3BNC117 (fig. S10A). However, when we evaluated plasma at the time of ART discontinuation or 4 weeks after, all treated RMs displayed limited ADCC to SHIV-AD8. Matched plasma from treated or control RMs did not exhibit SIVmac239-specific ADCC. The one exception was a single control RM with a non-specific response to target cell antigens, reflected by reactivity to both SHIV-AD8− and SIVmac239–infected targets (fig. S10B).

We next evaluated autologous neutralization responses using plasma collected from Study 2 at 5 and 17 weeks after ART discontinuation. These samples were interrogated by two different neutralization assays using HIV envelope (Env) pseudovirus or the SHIV-AD8 challenge virus. The median neutralizing ID_50_ titers were low in all RMs, and there was no significant difference in titer between treated and control RMs (fig. S10, C and D).

## Vaccinal effect of N-803 and bNAb combination

**Fig. 4.**
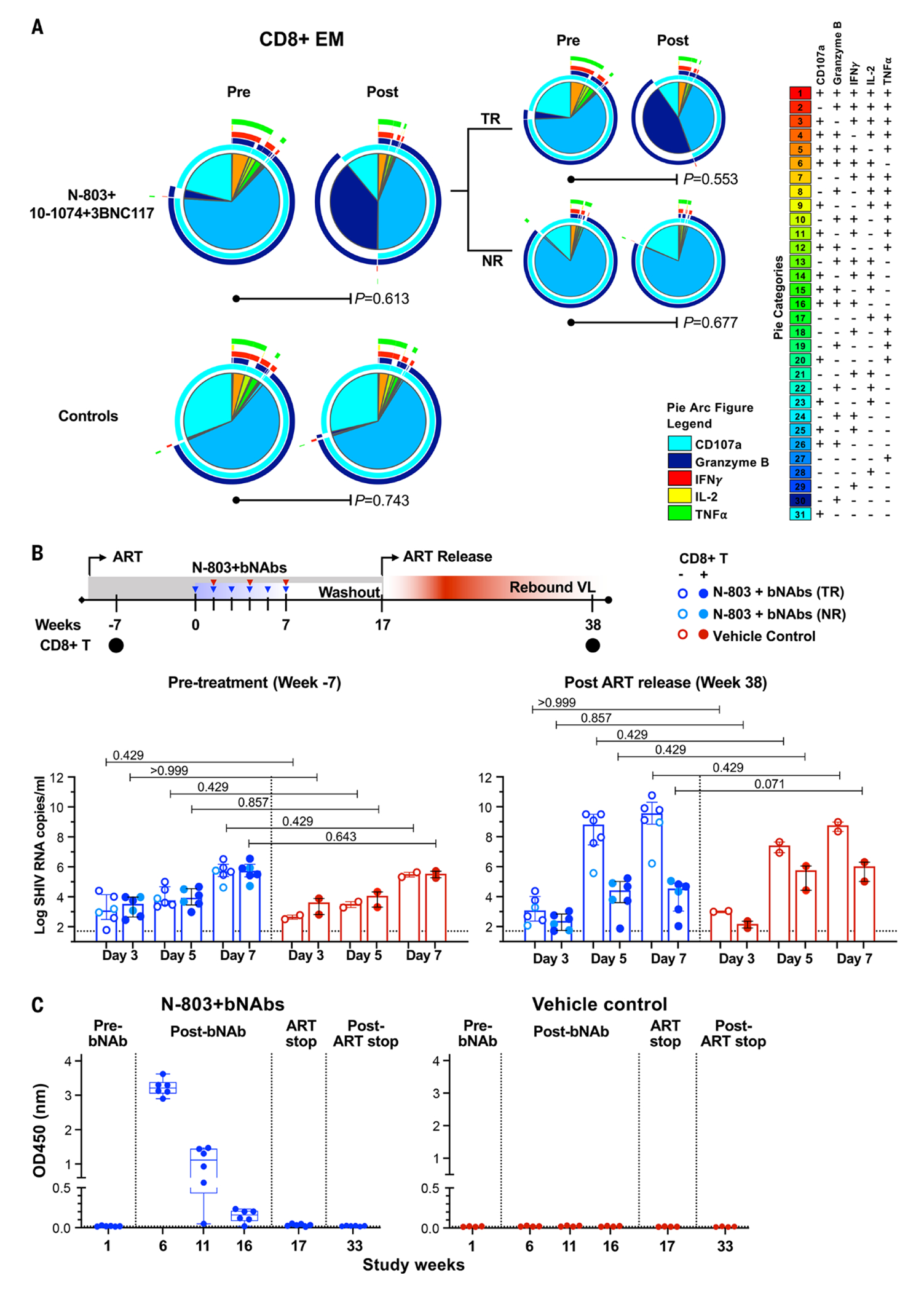
Modified CD8^+^ T cell effector function and bNAb-antigen binding. (**A**) Polyfunctional CD8^+^ T cell responses to SIVmac239 Gag peptides were measured by intracellular cytokine staining for CD107a, granzyme B, IFNγ, IL-2, and TNFα. The pie charts show the mean frequency of cells with different combinations of effector functions. Eight animals in N-803 treated group were then divided into treated-responder (TR, n=6) and treated non-responder (NR, n=2). *P*-values were computed using a permutation test described in Materials and Methods. (**B**) Ex vivo SHIV suppression assay to evaluate virus specific CD8^+^ T cell responses. Shown are viral replication in CD4^+^ T cells cultured alone or in the presence of autologous CD8^+^ T cells isolated from N-803+bNAb-treated animals that remained viremic or became viremic and control group. CD8^+^ T cells were isolated prior to treatment (Week −7) and 149 days after ART release. SHIV RNA copies in culture supernatant between CD4^+^ alone or with CD8^+^ T cells was measured by real-time PCR on days 3, 5, and 7 post culture. Statistical significance in differences in SHIV RNA with or without CD8^+^T cells was determined using a Kruskal–Wallis *H* test with Dunn’s post-test for multiple comparison. (C) Binding antibodies to BG505 SOSIP.664-His-gp140 protein were evaluated using samples isolated from treated (n=6) and control (n=4) RMs at pre-treatment (24 hours before the first bNAb dose), after bNAbs infusions (1, 5, and 11 weeks after the third N803 dose), ART discontinuation, and 16 weeks after ART discontinuation. Antibodies to BG505 SOSIP.664 trimer (1:100 diluted) at each representative time point are shown. The lower limit of detection is indicated as a hatched line. Box-and-whisker plot with all data points are shown. The binding of two bNAbs used in the study, 3BNC117 and 10-1074 to BG505 SOSIP.664-His-gp140 was tested. The average values and standard deviation of the mean from three separate experiments are shown.

We next evaluated CD8^+^ T cell responses by intracellular cytokine staining (ICS) following stimulation with SIV Gag peptides. We noted significantly enhanced post-treatment functionality as defined by high granzyme B, IFNγ, or TNFα expression in CM subsets (*P*=0.036) (fig. S11A). Controls did not show altered SIV-specific responses. Although SIV-specific IFN-γ and TNF-α expression significantly increased in CD8^+^ T EM populations following treatment, granzyme B did not appear different between treated and controls groups (fig. S11B). However, when we separated treated RMs into two groups (i.e., the six aviremic treatment responders (TR) and the two viremic non-responders (NR), respectively), we found that TRs displayed enhanced post-treatment granzyme B production, in addition to other cytokines (Fig. 4A). The NR RMs did not change their expression profiles after treatment, nor did they appear to differ from controls in this regard.

We next applied mathematical modeling to try to better understand the effect of N-803+bNAbs on CD8^+^ T cells. Between groups, neither N-803 nor bNAb alone enhanced viral suppression, but there was synergistic increase in effector function in N-803+bNAb–treated RMs compared with controls. Effector function for the N-803+10-1074 group was increased by 3.2-fold (*P*=0.11), whereas suppressive activity for the N-803+10-1074+3BNC117 group was 12.6 times higher and highly significant by the Wald test (*P*=9.9×10^−14^) (fig. S12, A and B).

To confirm N-803+bNAb–treated CD8^+^ T cells could independently contribute to enhanced viral control, we conducted an ex vivo viral suppression assay (20). Autologous SHIV-infected CD4^+^ T cells were mixed with unstimulated purified CD8^+^ T cells from RMs at 7 weeks before treatment, or at 38 weeks post-treatment (Fig. 4B). CD8^+^ T cells before treatment showed weak suppressive activity. However, CD8^+^ T cells isolated from the six TR RMs showed enhanced activity and reduced viral RNA by a median log 3.78 RNA copies/ml (range 1.78-7.22) on day 5 and median log 5.26 RNA copies/ml (range 1.87-7.75) on day 7 after culture as compared to CD4^+^ T cell culture alone (Fig. 4B). There was no such reduction in viral RNA from CD8^+^ T cells isolated prior to treatment (median log reduction 0.27, range −1.02-0.37 on day 5 and 0.003, range −0.19-0.17 on day 7).

Finally, we sought to understand if the modification of CD8^+^ T cells was due to bNAbs aiding the formation of antigen–anti-body complexes. We measured the capacity of antibodies in the plasma of treated or control RMs to bind BG505 SOSIP.664-His-gp140 trimers at various time points, including pre-treatment, 1, 6, and 11 weeks after the third bNAb dose, the time of ART discontinuation, and 16 weeks after ART discontinuation. In treated RMs, we observed significant BG505-trimer binding from plasma isolated 1 week after the third bNAb dose, which slowly declined over time to negative by the time of ART discontinuation (Fig. 4C). No BG505-trimer binding was detected in matched plasma samples from controls.

## Discussion

N-803, 10-1074, and 3BNC117 have been independently shown to have potent activity in RMs and PLWH (8, 11–13, 15, 16, 21, 22). Data from two independent studies demonstrate that N-803+bNAb(s) can induce long-term control of viremia after ART discontinuation in SHIV-AD8–infected RMs. Combining outcomes from each study, nine of 13 animals (~70%) treated with N-803+bNAbs maintained remission for ~10 months in Study 1 and ~8 months in Study 2.

During the treatment period, there was potent N-803-mediated induction of transient viremia (Fig. 2A and figs. S2 and S6), which has also been reported in phase 1 clinical studies (9). Based on our present data, N-803 treatment alone may not be sufficient to significantly alter clinical outcomes after ART discontinuation (Fig. 2B).

Our in vivo CD8^+^ cell depletion studies (Fig. 3E), combined with the paucity of ADCC and neutralizing responses (fig. S10, A to D), point to a role for CD8^+^ T cells in the suppression of viral rebound after ART discontinuation. Indeed, prior studies have shown that 10-1074 and 3BNC117 treatment of PLWH or viremic SHIV-infected RMs enhances the CD8^+^ T cell–mediated viral suppression in a portion of subjects (11, 12, 16, 17). However, the establishment of viral control in these studies was slow (up to 20 weeks) in RMs receiving bNAbs on day 3 PI, or up to 30 weeks in RMs receiving bNAbs on day 14 PI (11, 12). The viral control in our studies manifested rapidly and demonstrated that CD8^+^ T cell function can be reprogramed under ART suppression (Figs. 2B and 3B).

Our data indicate that N-803+bNAbs synergize to enhance CD8^+^ T cell function (Fig. 4A and fig. Sll, A and B). This immune profile is similar to one described in a case report of a PLWH with post-treatment control, expressing high granzyme B (23). Our modeling analyses also support the notion of enhanced CD8^+^ T cell function, but only when N-803+bNAb(s) are delivered in combination, and particularly when multiple bNAbs are used (fig. S12).

One possible mechanism for our observations involves viral control through ADCC. In our model, however, ADCC appears to be limited to when bNAbs are detectable (fig. S11, A and B). A second plausible scenario is that repeated episodes of transient viremia induced by N-803 result in bNAb–SHIV immune complexes, which are then available to APCs, resulting in a vaccinal boosting effect (16, 21, 22). As described above, a vaccinal effect has been reported in various scenarios using 10-1074 and 3BNC117 where antigen is readily available (11, 12, 14, 17). Similar outcomes and short-term viral control have been reported using structured ART interruption in a SHIV-RM model (24). Our data indicate that bNAbs may foster superior antigen–antibody complex formation over autologous antibodies in our SHIV-RM model (Fig. 4C). Thus, CD8^+^ lymphocytes play a central role in the control of viremia after ART discontinuation in N803+bNAb–treated SHIV-infected RMs. This CD8^+^ T cell-mediated control may include direct killing of infected, reactivated CD4^+^ T cells, as suggested by increased granzyme B expression, but also transcriptional silencing of HIV expression (8, 25–27).

Although significant differences exist between SHIV-infected RMs and PLWH, we sought to replicate the HIV reservoir in PLWH as closely as possible. Therefore, we choose a stringent SHIV-AD8 isolate known to have a reproducible viral rebound as compared to other SHIV isolates (28). Moreover, we initiated ART during the chronic stage of infection when viral reservoirs and host immunity are established; a situation that broadly parallels the timing of clinical identification and initiation of ART for many PLWH. Thus, our model seeks to recapitulate clinical HIV scenarios, and may present a higher threshold to viral control than studies using extremely early initiation of ART or SHIV isolates with high rates of spontaneous control. In conclusion, we demonstrate that complete eradication of the replication-competent viral reservoir is not a prerequisite for sustained remission after the discontinuation of ART. Furthermore, we find that immune-mediated control of viral rebound is achievable, sufficient, and sustainable in a stringent model of HIV infection on ART.

## Supplementary Material

supplemental materials

## Data Availability

All data are available in the main text or the supplementary materials.
