# Supplementary material for: Induction of durable remission by dual immunotherapy in SHIV-infected ART-suppressed macaques
Source: Science. Author manuscript; Available in PMC 2024 Apr 17. (PMC11022498; doi:10.1126/science.adf7966)
Supplement: supplemental materials [file NIHMS1984029-supplement-supplemental_materials.pdf]

Science AAAS

Supplementary Materials for

Induction of durable remission by dual immunotherapy in SHIV-infected ART-suppressed macaques

So-Yon Lim *et al.*

Corresponding author: James B. Whitney, whitnejc@bc.edu

DOI: 10.1126/science.adf7966

The PDF file includes:

Materials and Methods
Figs. S1 to S12
Tables S1 to S3
References

Other Supplementary Material for this manuscript includes the following:

MDAR Reproducibility Checklist (.pdf)

Materials and Methods

Animals

Thirty-six outbred 4-5-year-old specific pathogen–free (SPF) rhesus monkeys (35 males and 1 female) were used in Studies 1 (n=20) and 2 (n=16). All animals were screened to exclude the MHC class I alleles *Mamu-A*01*, *B*08*, and *B*17* associated with enhanced virologic control (*29*-*31*). Monkeys were housed at the Wisconsin National Primate Research Center (WNPRC). Monkeys were maintained according to the guidelines of the NIH Guide to the Care and Use of Laboratory Animals. The animal studies described were approved by the Wisconsin National Primate Research Center Institutional Animal Care and Use Committee.

SHIV infection and ART regimen

Animals were infected with 500 tissue culture infective doses ($TCID_{50}$) of SHIV-AD8, as described (*32*). The ART regimen, daily subcutaneous administration (1 ml per kilogram of body weight) of preformulated antiretroviral cocktail (5 mg/ml of tenofovir disoproxil fumarate (TDF), 40 mg/ml of emtricitabine (FTC), and 2.5 mg/ml of dolutegravir (DTG)) was initiated on day 52 (Study 1) and day 56 (Study 2) post infection and continued for 72 (Study 1) and 69 (Study 2) weeks (*33*). Animals were assigned to groups on the basis of age, body weight, viral load at study entry, SHIV RNA copies/ml on ART, AUC, $CD4^+$ and $CD8^+$ T cell counts, and $CD4^+/CD8^+$ T cell ratios across groups.

N-803 and bNAb treatment

In Study 1, Groups 1, 2, and 3 (n=5 per group) received N-803 alone, the V3 glycan supersite on HIV-1 Env-binding bNAb 10-1074 alone, or N-803 in combination with 10-1074, respectively. The control group (n=5) received vehicle. N-803 was dose-escalated in 10 RMs (Groups 1 and 3) with subcutaneous (SC) administration every week. The initial two doses of N-803 were 25 µg per kilogram of body weight followed by two doses at 50 µg per kilogram of body weight, a dose at 75 µg per kilogram of body weight and then a dose at 100 µg per kilogram of body weight. Two doses of 10-1074 at 10 mg per kilogram of body weight were administered in 10 RMs (groups 2 and 3) intravenously at 24 hours after the fourth and fifth N-803 doses. In Study 2, treatment group (n=8) received N-803 in combination with two bNAbs, the V3 glycan supersite on HIV-1 Env-binding bNAb 10-1074 and a bNAb specific for the CD4-binding site of HIV-1 Env (3BNC117). N-803 was dosed at the same concentration and frequency as in Study 1. Three doses of 10-1074 and 3BNC117 (10 mg of each antibody per kilogram of body weight) were administered every other week when the first dose of bNAbs was administered 24 hours after the second N-803 dose. The control group (n=8) received vehicle. At 70 days (Study 1) and 84 days (Study 2) after the last dose of 10-1074 and/or 3BNC117, ART was discontinued in all groups. Blood samples were collected at 24, 48, and 72 hours after each dose to monitor activation of immune cell subsets. Tissues including lymph node and colorectal mucosa were biopsied prior to the first treatment and after the last dose from each group of monkeys. After cessation of ART, 3 ml of blood was collected and processed over Ficoll on days 3, 7, and 10 and then weekly to monitor viral rebound. We included all animals in all analyses and confirmed all experimental findings were reliably reproduced. Technical staff were blinded during data acquisition and unblinded for analysis.

Lymphocyte staining and analysis

For Study 1, 100 µl of whole blood was stained with following antibody panels: NK cell panel: anti-CD3 (SP34.2), anti-CD8 (SK1), anti-NKG2A (Z199, Beckman Coulter), anti-CD16 (3G8), anti-CD56 (NCAM16.2), anti-CD69 (TP1.55.3, Beckman Coulter), anti-NKG2D (BAT221, Miltenyi Biotec), and anti-NKp30 (REA823, Miltenyi Biotec); T cell panel: anti-CD3, anti-CD4 (L200), anti-CD8, anti-CD20 (2H7), anti-CD25 (M-A251), anti-CD28 (CD28.2, Beckman Coulter), anti-CD69, and anti-CD95 (DX2). Cells were treated with BD Pharm Lyse (BD Bioscience) for RBC lysis, fixed with 2% paraformaldehyde. For Study 2, PBMCs were isolated by Ficoll density gradient centrifugation. Approximately 5×10^5 PBMCs were stained with each of the following panels of antibodies: NK cell panel: anti-CD3, anti-CD8, anti-NKG2A, anti-CD16, anti-CD20, anti-CD56, anti-CD69, anti-NKG2D, anti-NKp30 and anti-NKp46; T cell panel: anti-CD3, anti-CD4, anti-CD8, anti-CD20, anti-CD25, anti- CD28, anti-CD69, anti-CD95. All antibodies are from BD Biosciences unless otherwise indicated. More detailed information on antibodies used is shown in table S3.

Lymphocyte phenotyping analysis

Cells were treated with ACK lysis buffer (Gibco) for further RBC lysis, fixed with 2% paraformaldehyde. Stained cells were acquired on a FACS LSRII flow cytometer (BD Biosciences), and analyzed using FlowJo Software v. 10 (FlowJo, LLC, Ashland, OR). All events were gated first on FSC singlets and then lymphocytes. NK cells were identified as $CD3^{-}CD8^{+}NKG2A^{+}$, and CD16 and CD56 were used to define subsets. $CD4^{+}$ and $CD8^{+}$ T cells were identified as $CD3^{+}CD20^{-}$, and CD28 and CD95 were used to define subsets (*34*). All activation markers, including CD25 and CD69, were measured as a percent of lymphocyte population of interest.

bNAb concentration in plasma and PK analysis

Plasma levels of the bNAbs, 3BNC117 and 10-1074 were determined using a validated luciferase-based neutralization assay in TZM-bl cells as described (*35*). Briefly, plasma samples were tested using a primary 1:20 dilution with a fivefold titration series against HIV-1 Env pseudoviruses Q842.d12 and X2088_c9, which are highly sensitive to neutralization by 3BNC117 and 10-074, respectively. Standard curves were generated using 3BNC117 and 10–1074 clinical drug products and included in every assay set-up using a primary concentration of 10 µg/ml with a fivefold titration series. Plasma concentrations of 3BNC117 and 10–1074 for each sample were then calculated as follows: plasma ID_{50} titer (dilution) × 3BNC117 IC_{50} or 10–1074 IC_{50} titer (µg/ml) = plasma concentration of 3BNC117 or 10–1074 (µg/ml).

$CD4^{+}$ T cell cytometric sorting

Cryopreserved samples of PBMC, LMNC, and GMMC biopsies were thawed at 37°C in RPMI 1640 containing 10% fetal bovine serum (FBS) and benzonase (Millipore) at 25 U/ml. Cells were resuspended in 1X phosphate-buffered saline (PBS) containing LIVE/DEAD Fixable Aqua Dead Cell Stain (Life Technologies) for 20 min at room temperature in the dark. Cells were washed and stained with the following fluorescently conjugated antibodies: PBMCs and LN: anti-CD3 (SP34.2), anti-CD4 (L200), anti-CD8 (SK1), anti-CD20 (2H7), anti-CD28 (CD28.2), anti-CD45 (D058-1283), anti-CD95 (DX2), anti-CCR7 (150503), anti-ICOS (C398.4A, Biolegend), anti-PD-1 (EH12.2H7, Biolegend), anti-CXCR3 (1C6/CXCR3), and anti-CXCR5 (MU5UBEE, Thermo Fisher); GMMC: anti-CD3, anti-CD4, anti-CD8, anti-CD28, anti-CD45, anti-CD95, and anti-CCR7. After 15 min in the dark, cells were washed, resuspended in ice-cold 1X PBS, and were sorted using a FACS Aria II (BD Bioscience). Sorted $CD4^{+}$ T cells were FSC singlets, live,

$CD45^+CD3^+CD4^+CD8^−$ lymphocytes. Subsets were defined as follows: naïve ($CD95^−CD28^+CCR7^+$), central memory ($CD95^+CD28^+CCR7^+$), transitional memory ($CD95^+CD28^+CCR7^−$), follicular helper T cells ($CD95^+CD28^+CXCR5^{hi}PD\text{-}1^{hi}$), and effector memory ($CD95^+CD28^−$). More detailed information on antibodies used is shown in table S3.

Integrated DNA and intact proviral DNA assay (IPDA)

Genomic DNA was isolated from sorted $CD4^+$ T cells using either a QIAamp DNA blood mini kit or QIAamp DNA micro kit following Manufacturer's instruction. To detect integrated SHIV DNA, we applied the published methods modified for rhesus macaque specific Alu sequences as described (*33*, *36*). Genomic DNA was serially diluted in 10-fold dilutions (1000 to 1 cell equivalents) and applied to the first-round PCR. SIV LTR nested PCR products were then subjected to a standard qRT-PCR. Cell numbers analyzed in each reaction was confirmed by simultaneous q-PCR of GAPDH genes. A cloned cell line, 3D8 containing a single integrated copy of SIV DNA was used an integration standard. Intact SHIV proviral DNA was quantitated using a novel droplet digital PCR-based method designed to detect intact SHIV genomes, called the SHIV Intact Proviral DNA Assay (IPDA). This assay uses principles similar to those previously described for the analysis of intact HIV-1 and SIV proviruses and includes corrections for 2LTR circles and DNA shearing as described (*37*, *38*). The 3′ SHIV IPDA amplicon was the same as the 3′ HIV-1 amplicon, whereas the 5′ amplicon was in the gag gene.

In vivo $CD8^+$ lymphocyte depletion

Rhesus macaques were depleted of $CD8^+$ lymphocytes using the MT807R1 monoclonal antibody (Nonhuman Primate Reagent Resource) using the previously described protocol (*39*) CD4 and CD8 lymphocytes were monitored twice weekly for the first 3 weeks and then weekly throughout the monitoring period. Whole blood was stained with the antibodies anti-CD4 (L200), anti-CD8 (DK25, Dako), and anti-CD3 (SP34.2). The anti-CD8 antibody clone DK25 was used for detection, as described (*40*). RBCs were lysed using a TQ-Prep (Beckman Coulter), fixed in 2% formaldehyde, acquired on a FACS Calibur (BD Biosciences), and analyzed using FlowJo Software. All antibodies are from BD Biosciences unless otherwise indicated. More detailed information on antibodies used is shown in table S3.

Intracellular cytokine staining

The measurement of intracellular cytokines (TNF-α, IFN-γ, IL-2, granzyme B, and CD107a) was performed as previously described (*41*). Thawed PBMCs were rested at 37°C for 3 hours before stimulation. Approximately 3×10^6 cells were stimulated with either dimethyl sulfoxide alone (unstimulated) or overlapping peptide pools (2 μg/ml) covering the entire SIVmac239 Gag protein (NIH AIDS Reagent Program, catalog no. 6204) and anti-CD49d antibodies (1 μg/ml; BD Biosciences). Cells were incubated with anti-CD107 antibody (H4A3) for 6 hours, with GolgiStop (BD Bioscience) and GolgiPlug (BD Bioscience) added 1 hour after stimulation. Cells were stained with LIVE/DEAD Fixable Aqua Dead Cell Stain (Invitrogen) followed by antibodies conjugated for surface staining: anti-CD4 (L200), anti-CD8 (SK1), anti-CD28 (L293), anti-CD45 (D058-1283), and anti-CD95 (DX2). Cells were permeabilized with BD Cytofix/Cytoperm for 20 min followed by intracellular staining using: anti-CD3 (SP34.2), anti-CD69 (TP1.55.3, Beckman Coulter), anti-TNF-α (Mab11), anti-IFN-γ (B27), anti-IL-2 (MQ1-17H12), and anti-granzyme B (GB-11). All antibodies are from BD Biosciences unless otherwise indicated. More detailed information on antibodies used is shown in table S3. The value of percent cytokine-positive cells in unstimulated samples was subtracted from the corresponding value of stimulated samples.

Antibody-dependent cellular cytotoxicity (ADCC)

ADCC activity was measured as described (*42*). CEM.NKR-$_{CCR5}$-sLTR-Luc target cells were infected with VSV G-pseudotyped SHIV-AD8Δvif or SIVmac239Δvif by spinoculation in the presence of 40 μg/ml of Polybrene. Two days post infection, infected cells were incubated with diluted plasma and an CD16 expressing NK cell line at a 10:1 effector-to-target ratio for 8 hours. The loss of Luc activity was used as to indicate antibody-mediated killing of infected cells. Infected target cells incubated with NK cells in the absence of antibody were used to measure maximal Luc activity and uninfected target cells cultured with NK cells were used to determine background.

TZM.bl pseudovirus neutralization assay

Virus neutralization was measured using a luciferase-based assay in TZM.bl cells as previously described (*32*). Briefly, plasma dilutions were performed in duplicate using 96-well flat-bottom plates. Two hundred $TCID_{50}$ of SHIV-AD8 was added to each well prior to incubation for 1 hour at 37°C. TZM.bl cells were seeded at 1×10^4 cells per well with D-MEM media containing DEAE-dextran (Sigma, St. Louis, MO) at a final concentration of 11 μg/ml. After a 48-hour incubation, 150 μl of supernatant was removed and replaced with 100 μl of Bright-Glo luciferase reagent (Promega, Madison, WI). Following lysis, 150 μl of the sample lysate was measured using a Victor 3 luminometer (Perkin Elmer). The 50% inhibitory dose (ID_{50}) titer was calculated as described. Data were analyzed with software provided by the CAVD Vaccine Immunology Statistical Center.

TZM.bl neutralization assay with SHIV-AD8 challenge stock

Neutralization against SHIV-AD8 was assessed as described (*43*) using TZM-bl cells and 4 ng of SHIV p27 per well. TZM-bl cells were seeded to flat-bottom 96-well plates the day before each neutralization assay at a density of 1.5×10^4 cells per well. Plasma dilutions and viruses were incubated for 1 hour at 37°C before being combined with reporter cells. Luciferase activity in TZM-bl cells was measured 3 days later using BriteLite Plus substrate (Perkin Elmer).

Viral Suppression Assay

Viral suppression assay was performed as described with some modifications (20). Briefly, frozen PBMC isolated animals at 3 days prior to SHIV infection were thawed and $CD4^+$ T cells were enriched using $CD4^+$ T Cell Isolation Kit NHP (Miltenyi). Enriched $CD4^+$ T cells were plated at 5×10^5 cells per well in 12-well plates and stimulated with 10 µg/ml of concanavalin A, (Sigma) and 20 U/ml of rhIL-2 (Roche via NIH AIDS reagent resource #136) in RPMI supplemented with 10% FBS (R10+) for 3 days. Activated $CD4^+$ T cells were harvested and subjected to a Ficoll gradient to remove dead cells and debris. Fraction of the activated $CD4^+$ T cells were taken for flow cytometry staining and from the rest distributed across 15-ml conical tubes at 1×10^5 cells per tube for infection. From the virus stock of 1.110^{-3} MOI, 25 µl was added to the falcon tube with 75 µl of $CD4^+$ T cells in R10+ media supplemented with 20 U per ml of IL-2 and incubated at 37°C for 3 hours with intermediate mixing. At the same time, $CD8^+$ T cells were isolated using $CD8^+$ T Cell Isolation Kit NHP (Miltenyi) from frozen PBMCs isolated 7 weeks prior to N-803+bNAb treatment (47 weeks after ART start) and 149 days after ART release from the same animals used above for $CD4^+$ T cells isolation. After 3 hours of infection, $CD4^+$ T cells were washed three times with R10+ media and plated with respective $CD8^+$ T cells to obtain CD4:CD8 ratios of 1:4, 1:2, and 1:1 and without $CD8^+$ T cells in $R10^+$ media supplemented with 20 U/ml of IL-2 in 96 well plate. On postinfection days 3, 5, and 7, plates were centrifuged for 1400*g* for 5 min and supernatant was collected for qRT-PCR analysis. Fresh $R10^+$ media supplemented with

20 U/ml of IL-2 was added to replenish the wells at respective time points. At day 7, cells were harvested and stained with LIVE/DEAD Fixable Aqua Dead Cell Stain (Invitrogen) followed by conjugated antibodies for surface staining: anti-CD4 (L200), anti-CD8 (SK1), anti-CD20 (2H7), anti-CD25 (M-A251), anti-CD28 (CD28.2), anti-CD95 (DX2), anti-HLD-DR (G46-6). Intracellular stain was performed by permeabilizing cells with BD Cytofix/Cytoperm for 20 min and stained with anti-CD69 (TP1.55.3, Beckman Coulter) and anti-CD3 (SP34.2). All antibodies are from BD Biosciences unless otherwise indicated. Infection rate was assessed by supernatant levels of virus by RT-qPCR.

Viral Dynamic Modeling

The observed viral load (VL) after treatment interruption showed a range of different trajectories (**fig. S12B**). Some were reminiscent of post-treatment control (PTC, e.g., rh2664), whereas others showed a typical peak VL followed by a VL set point (e.g., rh2668, rh2670). We used viral dynamic modeling to find a possible mechanistic explanation for these different patterns, and to relate them to the type of immunotherapy applied to each animal. The VL trajectories showing a set-point are well described by the standard viral dynamic model (*44*). However, other animals (e.g., rh2654) showed highly oscillatory VL dynamics, which are not well described by the standard mode. We therefore extended them by including an immune response that becomes exhausted at high antigen concentrations. This extension is known as the PTC model (*45*), which is shown schematically in fig. S6 and is formulated in terms of a system of ordinary differential equations (ODEs). These describe target cells (T, CD4 T cells susceptible to viral infection), infected CD4 T cells (I) that produce free virus V, and effector cells (E) that eliminate productively infected cells by cytotoxic and non-cytotoxic effects. The ODEs for T, I, V, and E are then given by:

$$\frac{dT}{dt} = \lambda_T - d_T T - \beta V T$$

$$\frac{dI}{dt} = \beta V T - d_I I - \kappa E I$$

$$\frac{dV}{dt} = pI - cV$$

$$\frac{dE}{dt} = \lambda_E - d_E E + b\frac{EI}{k_B + E + I} - \xi\frac{EI}{k_X + I}$$

In the first equation, target cells, *T*, are supplied at a constant rate λ_T, die at per capita rate d_T, and are infected at rate βV, where β is the infection rate constant. In the second equation, productively infected cells, *I*, die at per capita rate d_I, and are eliminated by effector cells by cytolytic or non-cytolytic processes at rate κE, where κ is the elimination rate constant. In the third equation, virions, V, are produced at rate p per infected cell and cleared at rate c per virion. Finally, in the fourth equation, effector cells enter at a constant rate λ_E, and die at per-capita rate d_E. When effector cells encounter infected cells, they proliferate at maximal rate b in a saturated manner, following previously published work (*46, 47*). Saturation is governed by the parameter k_B. Finally, the PTC model assumes that effector cells can become exhausted at high antigen concentrations at maximum rate ξ. The antigen concentration at which exhaustion is at its half-maximum is given by the parameter k_X.

We make the common assumption that the observed VL has a log-normal distribution with location parameter $\log(V)$, and scale parameter σ. VL measurements below the limit of detection of 50 copies per mililiter are treated as left-censored observations. We fit this model to the VL time series from all animals simultaneously using a non-linear mixed effects framework (**fig. S13**). In this method, it is assumed that each animal has unobserved individual parameters, which uniquely

determine the VL trajectory. We used Monolix 2021R2 (Lixoft SAS, a Simulations Plus company) to infer the distributions that gave rise to these individual-level parameters, and to sample from these distributions, conditional on the observed time series. As not all parameters are identifiable from observing merely the VL, we fix some parameters to biologically plausible values, namely $c = 23$ per day, $p = 1000$ per day and $d_I = 0.6$ per day. In addition, we compute the significance of the effect of the immunotherapy pre-ART interruption on the model's parameters using the Wald test.

Ni-NTA ELISA

Antibody binding to Env trimer experiment was performed as previously described (*48*). Briefly, Ni^{2+}-nitrolotriacetic acid (Ni-NTA) coated Hissorb 96-well plates (Qiagen) were coated with 200 ng per well of BG505 SOSIP.664-His-gp140 for 1 hour at RT. After washing unbound trimers away with 0.05% Tween-20-PBS (PBS-T), plates were incubated with plasma samples diluted 1:100 in 1% BSA-PBS and appropriate positive controls (3BNC-117 and 10-1074) at 4°C for ON. After washes with PBS-T, goat HRP-conjugated anti-human IgG (Southern Biotech) was added at 1:2000 dilution in 1% BSA-PBS and then incubated for 45 min at RT. Colorimetric detection was performed using TMB substrate (Pierce) and stopped using 0.8 M H_2SO_4 (ThermoFisher). Optical densities (OD) were measured at 450 nm using the PowerWave 340 microplate reader.

Statistical analyses

Analyses of virologic or immunologic data at study entry and comparisons of grouped variables were performed using a non-parametric Kruskal–Wallis *H* test to compare three or more unmatched groups or a Mann–Whitney *U* test to compare differences between two groups. Transformed log10 SIV RNA levels in plasma were calculated using GraphPad Prism (version 9.0). Changes in values after treatment within groups were analyzed by use of a Wilcoxon matched pairs signed rank test. *P*-values were adjusted for multiple comparisons when more than two groups were compared. All flow cytometry data in this manuscript was analyzed using FlowJo (v10). Background subtraction and formatting of exported data from FlowJo was performed with Pestle (version 2). Statistical analyses of the polyfunctionality and immune cell subset and display of multicomponent distributions were performed using the Simple Presentation of Incredibly Complex Evaluations software (SPICE 6.1). To compare each of the pies as calculated by SPICE, one-sided permutation tests were performed. *P*-values were considered significant if <0.05 after adjustment for multiple comparisons when applicable.

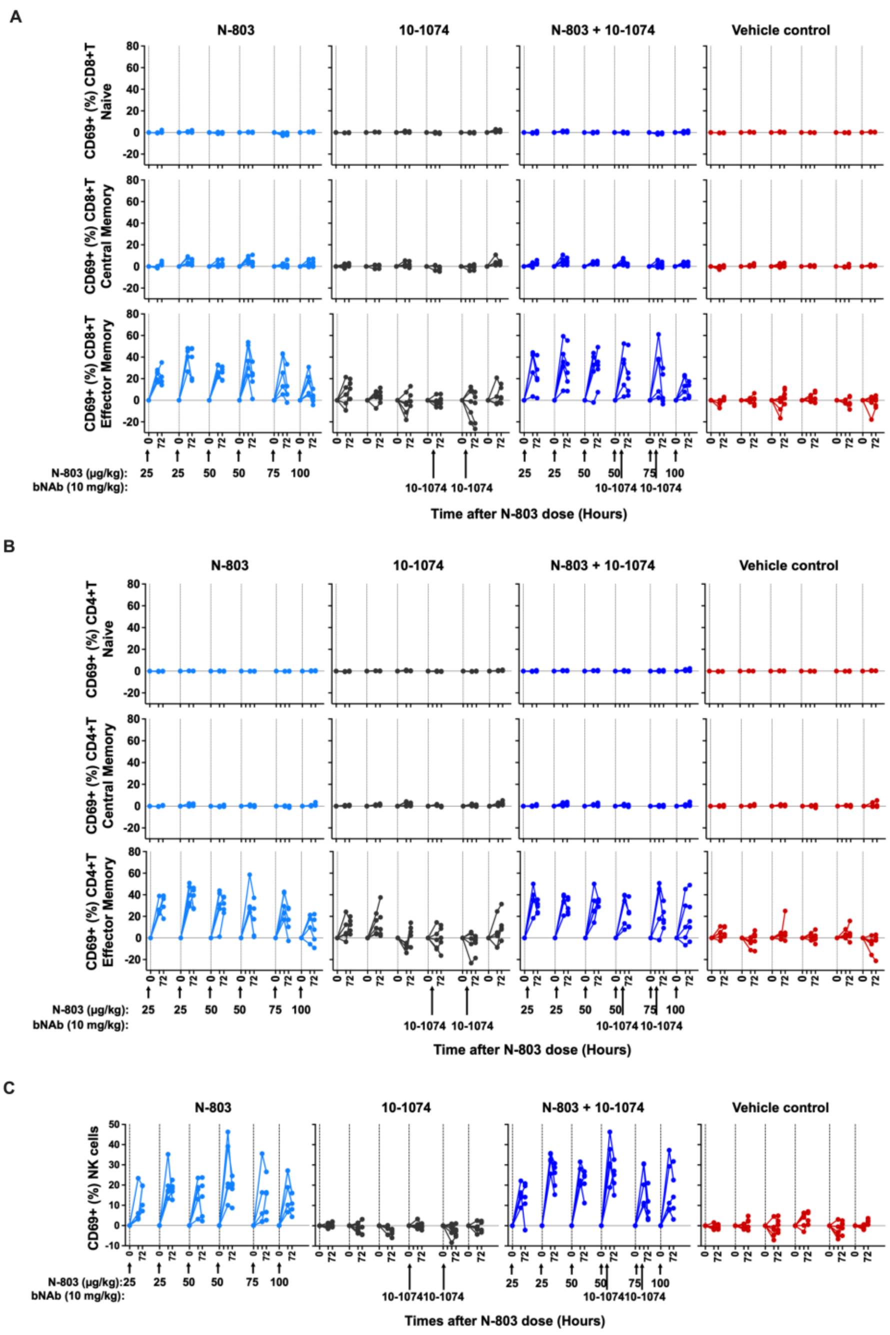

Fig. S1. **Activation of T and NK cells following N-803+10-1074 administration**. Activation of naïve ($CD95^{-}CD28^{+}$), central memory ($CD95^{+}CD28^{+}$), and effector memory ($CD95^{+}CD28^{-}$) populations by cytometric detection of CD69 in (**A**) $CD8^{+}$ T cells and (**B**) in $CD4^{+}$ T cells. CD69 expression within each subset was measured at the time of each dose (baseline) and at 48 and 72 hours after each N-803 dose. (**C**) Activation of NK cells were monitored by flow cytometric detection of CD69. Changes in CD69 expression are shown as the absolute difference in percent from the day of dose for all immune subsets. Both N-803 and 10-1074 dosing are indicated by black arrows on the *x*-axis.

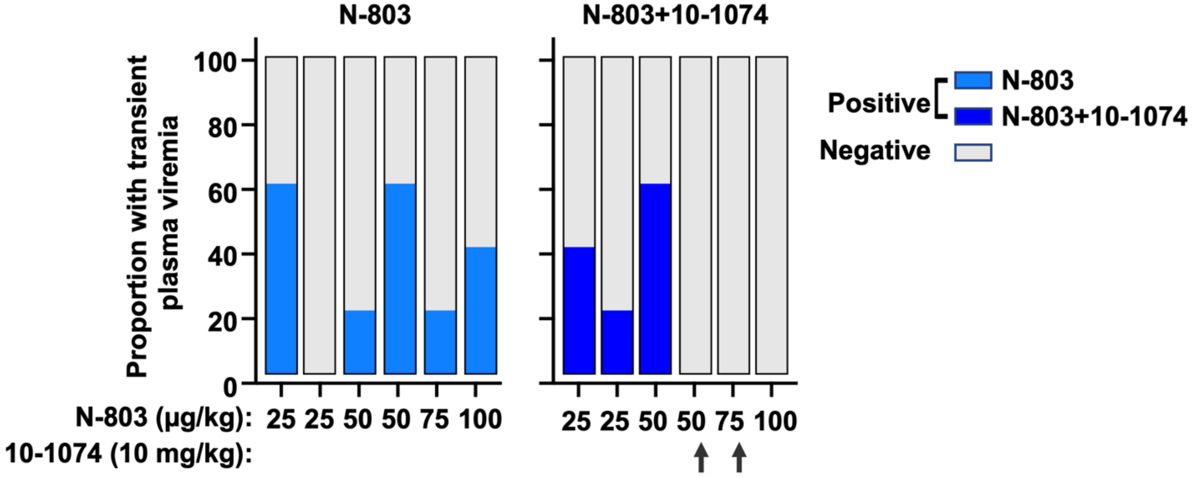

Fig. S2. Transient plasma viremia induced by N-803 dosing in SHIV-infected monkeys on ART. The proportion of RMs with detectable plasma viremia (>50 copies/ml) measured at 48 or 72 hours after each N-803 dose are shown. N-803 concentrations at each dose are indicated. Arrows indicate the timing of bNAb dosing at 24 hours after N-803 treatment.

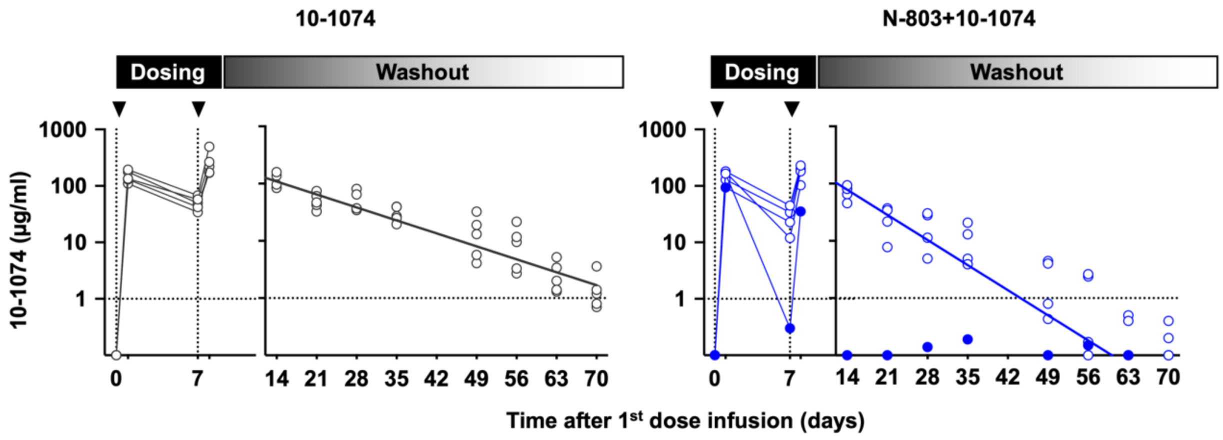

Fig. S3. Pharmacokinetics of 10-1074 in Study 1. The plasma concentration of 10-1074 was monitored during dosing (predose and 24 hours post dose) and during the washout period by TZM.bl assay. The RM that developed an ADA response is indicated by closed blue circles. Lines indicate the lower limit of accuracy of the assay (1 µg/ml).

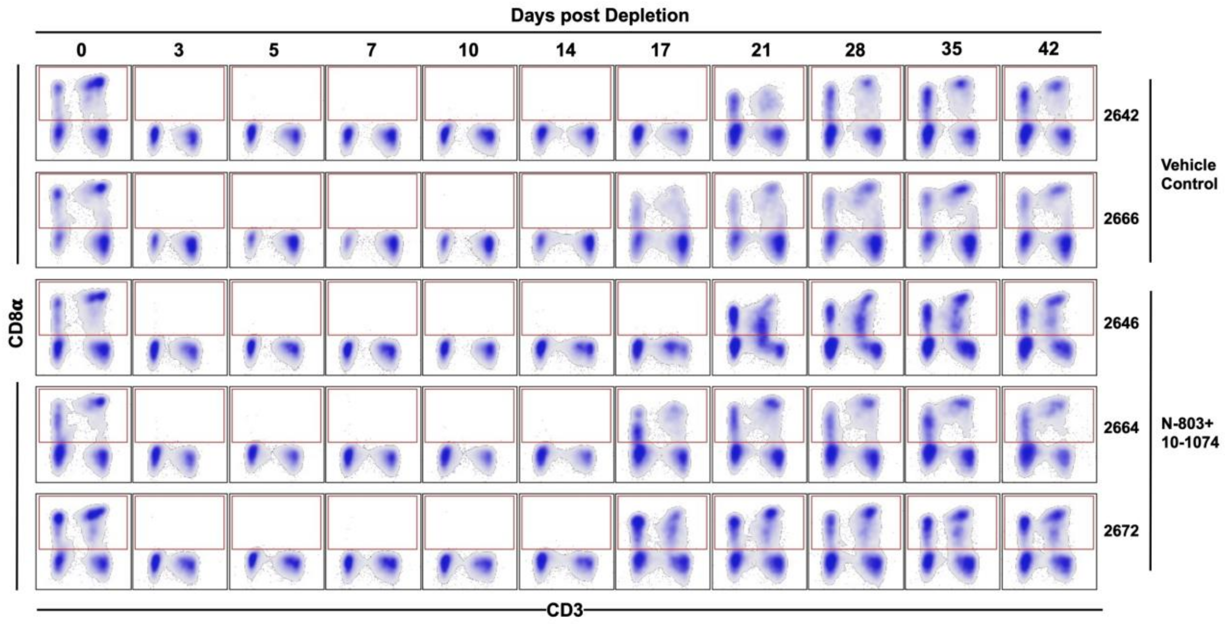

Fig. S4. In vivo CD8⁺ T cell depletion. CD8⁺ T cells were depleted in vivo using the monoclonal antibody MT807R1 in two control macaques and three macaques treated with N-803+10-1074. The presence of CD8⁺ T cells in the blood was monitored by flow cytometry.

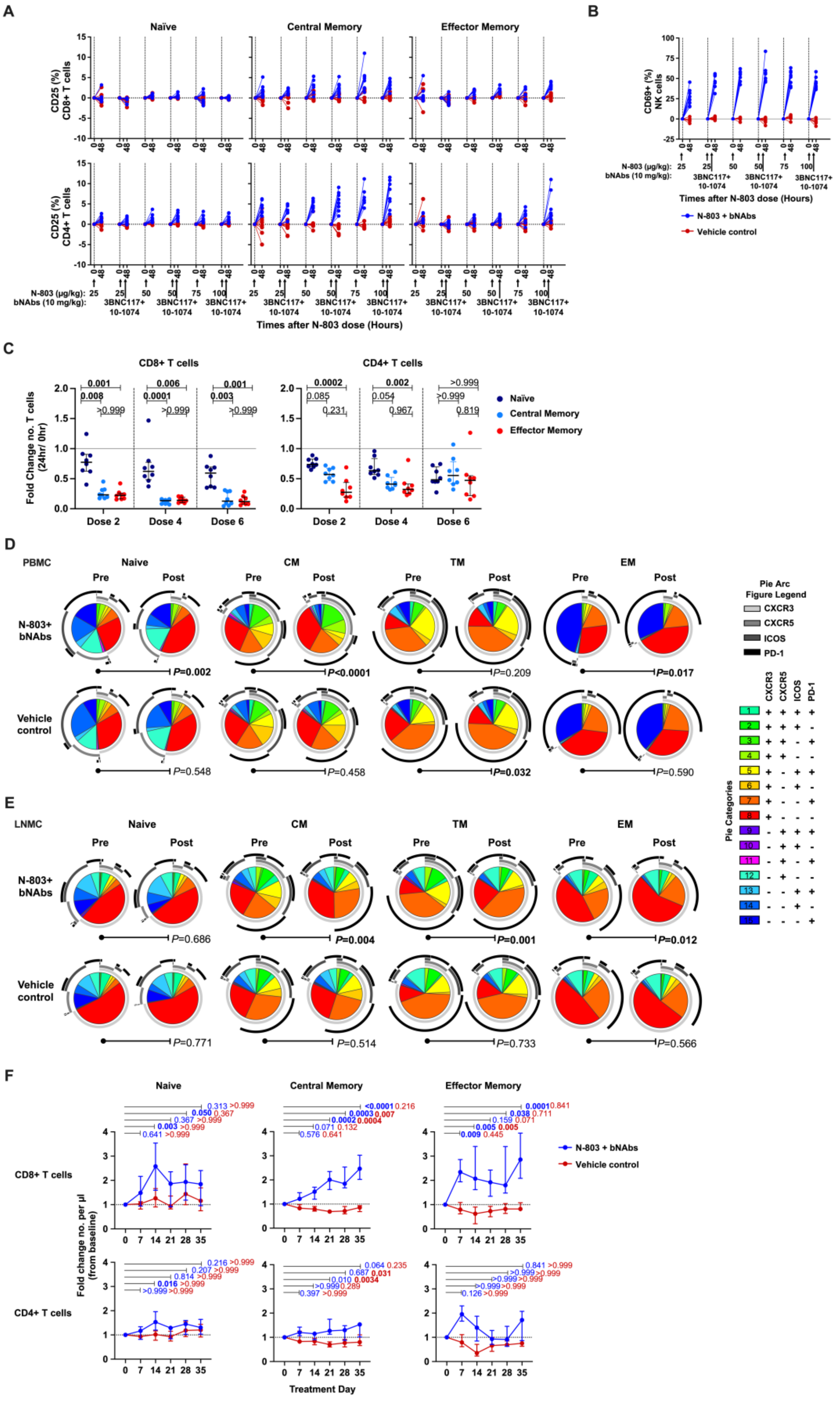
A
Naïve
Central Memory
Effector Memory
CD25 (%) CD8+ T cells
CD25 (%) CD4+ T cells
N-803 (µg/kg):
bNAbs (10 mg/kg):
3BNC117+ 10-1074
Times after N-803 dose (Hours)
B
CD69+ (%) NK cells
Times after N-803 dose (Hours)
N-803 + bNAbs
Vehicle control
C
CD8+ T cells
CD4+ T cells
Fold Change no. T cells (24hr/ 0hr)
Dose 2
Dose 4
Dose 6
Naïve
Central Memory
Effector Memory
D
PBMC
Naive
CM
TM
EM
Pre
Post
N-803+ bNAbs
Vehicle control
P=0.002
P<0.0001
P=0.209
P=0.017
P=0.548
P=0.458
P=0.032
P=0.590
Pie Arc Figure Legend
CXCR3
CXCR5
ICOS
PD-1
Pie Categories
E
LNMC
P=0.686
P=0.004
P=0.001
P=0.012
P=0.771
P=0.514
P=0.733
P=0.566
F
Naive
Central Memory
Effector Memory
CD8+ T cells
CD4+ T cells
Fold change no. per µl (from baseline)
Treatment Day
N-803 + bNAbs
Vehicle control

Fig. S5. Rapid activation, margination, and expansion of $CD8^+$ and $CD4^+$ T memory subsets and NK cells following N-803+10-1074+3BNC117 administration. In Study 2, the expression of CD25 in naïve ($CD95^-CD28^+$), central memory ($CD95^+CD28^+$), and effector memory ($CD95^+CD28^-$) subsets from $CD8^+$ and $CD4^+$ T cells was monitored by flow cytometry (**A**) Activation of NK cells were monitored by flow cytometric detection of CD69 (**B**) CD25 and CD69 expression within each subset was measured at the time of each dose (baseline) and at 48 hours after each N-803 dose. Changes in CD25 and CD69 expression are shown as the difference in percent from the day of dose for all immune subsets. Both N-803 and 3BNC117+10-1074 dosing is indicated by black arrows on the *x*-axis. Absolute counts of each memory subset of $CD8^+$ and $CD4^+$ T cells per microliter of blood were monitored by flow cytometry at the day of dose (baseline) and at 24 hours after doses 2, 4, and 6. The fold change in each memory subsets were calculated by the day of dose to 24 hours after each dose (doses 2, 4, and 6). Data shown as median ± interquartile range (n=8 per group) (**C**) Boolean-gated frequencies of markers (CXCR3, CXCR5, ICOS, nd PD-1) within FlowJo were exported into SPICE. Pie charts and arcs showing different combinations of trafficking or costimulation/activation markers in $CD8^+$ T memory subsets in (**D**) peripheral blood and (**E**) LMNC. *P*-values were computed using a permutation test. (**F**) Fold changes in each subset of $CD8^+$ and $CD4^+$ T cells were calculated and *P*-values comparing $CD8^+$ and $CD4^+$ T cell expansion at each treatment day versus day of dose (baseline) are shown for both treated (blue) and control (red) groups. Statistical analysis was performed using the Kruskal–Wallis *H* test followed by Dunn's test for multiple comparison (C) or a repeated measures two-way ANOVA with Geisser–Greenhouse correction for multiple comparison (F) as required. Adjusted *P*-values are shown.

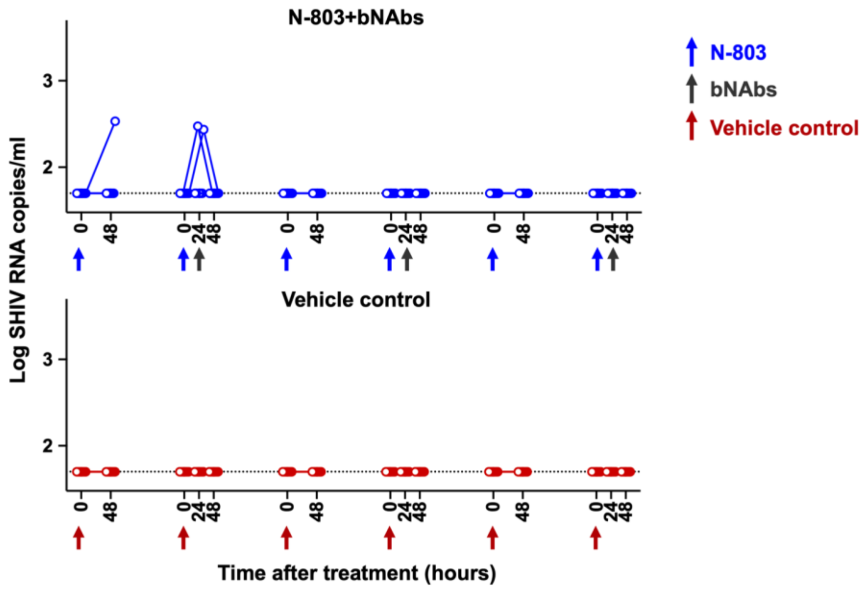

Fig. S6. Transient plasma viremia induced by N-803 dosing in SHIV-infected monkeys on ART in Study 2. SHIV RNA copies were monitored in RMs at baseline and 24 and 48 hours following each of six weekly N-803 doses in all treatment and control groups. Arrows indicate the timing of N-803+10-1074+3BNC117 or vehicle dosing.

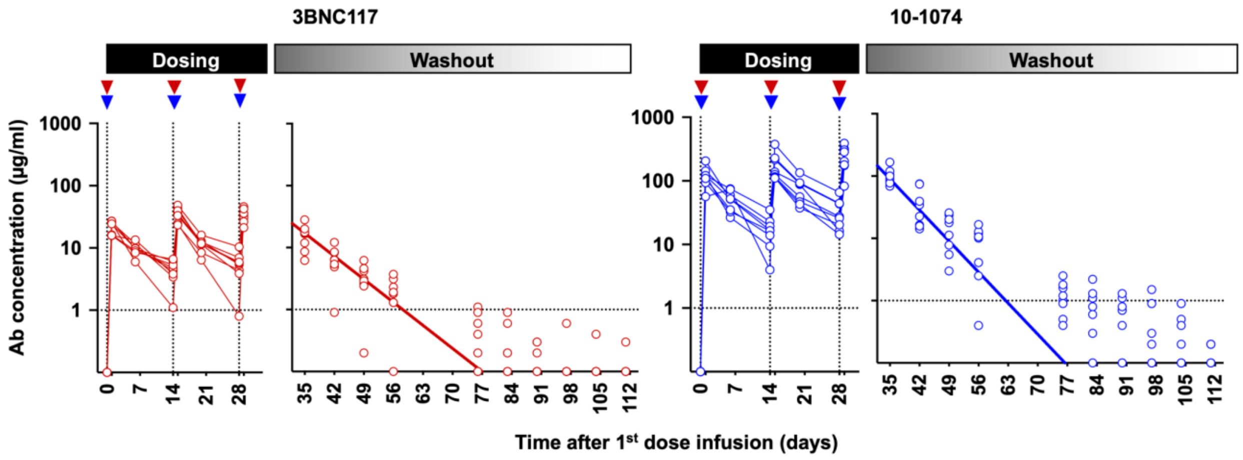

Fig. S7. Pharmacokinetics of 10-1074 and 3BNC117. In Study 2, plasma concentrations of 3BNC117 and 10-1074 were monitored during dosing (0 and 24 hours post dose) and during the washout period by TZM.bl assay.

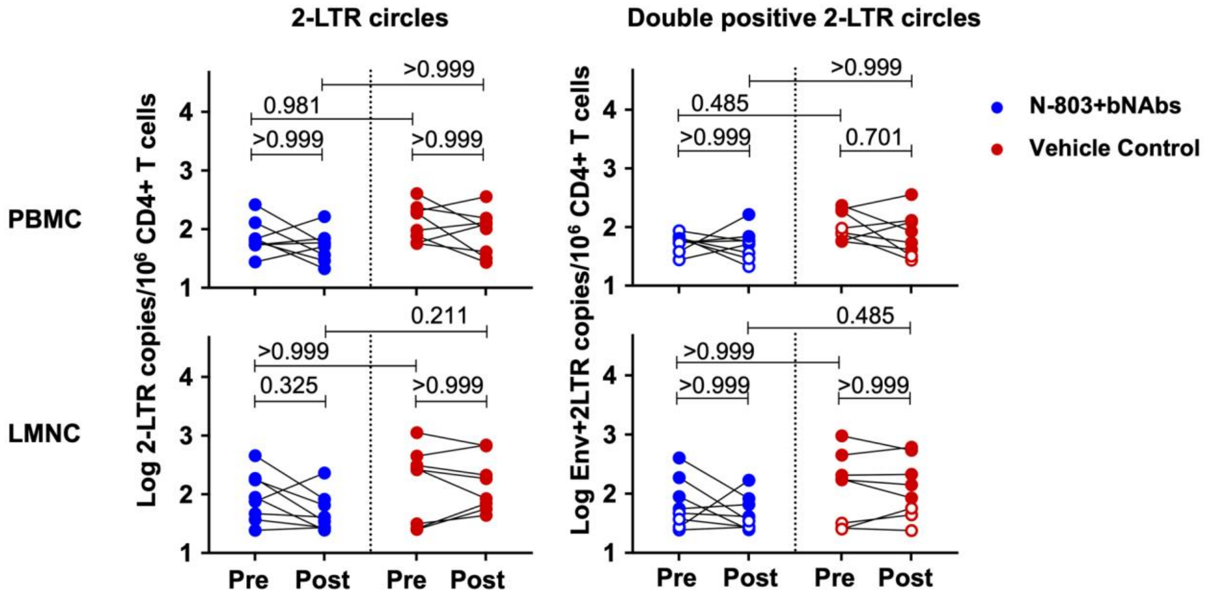

Fig. S8. Comparisons of 2-LTR circles in CD4+ T cells before and after treatment. Levels of 2-LTR circles are expressed as log10 copies per 10^6 $CD4^+$ T cells. 2-LTR circles and double positive 2-LTR circles were compared between groups using a Kruskal–Wallis *H* test at pre- and post-treatment timepoints. Values within each group were compared using a Wilcoxon matched-pairs signed-rank test. *P*-values were adjusted for multiple comparison by Dunn's test.

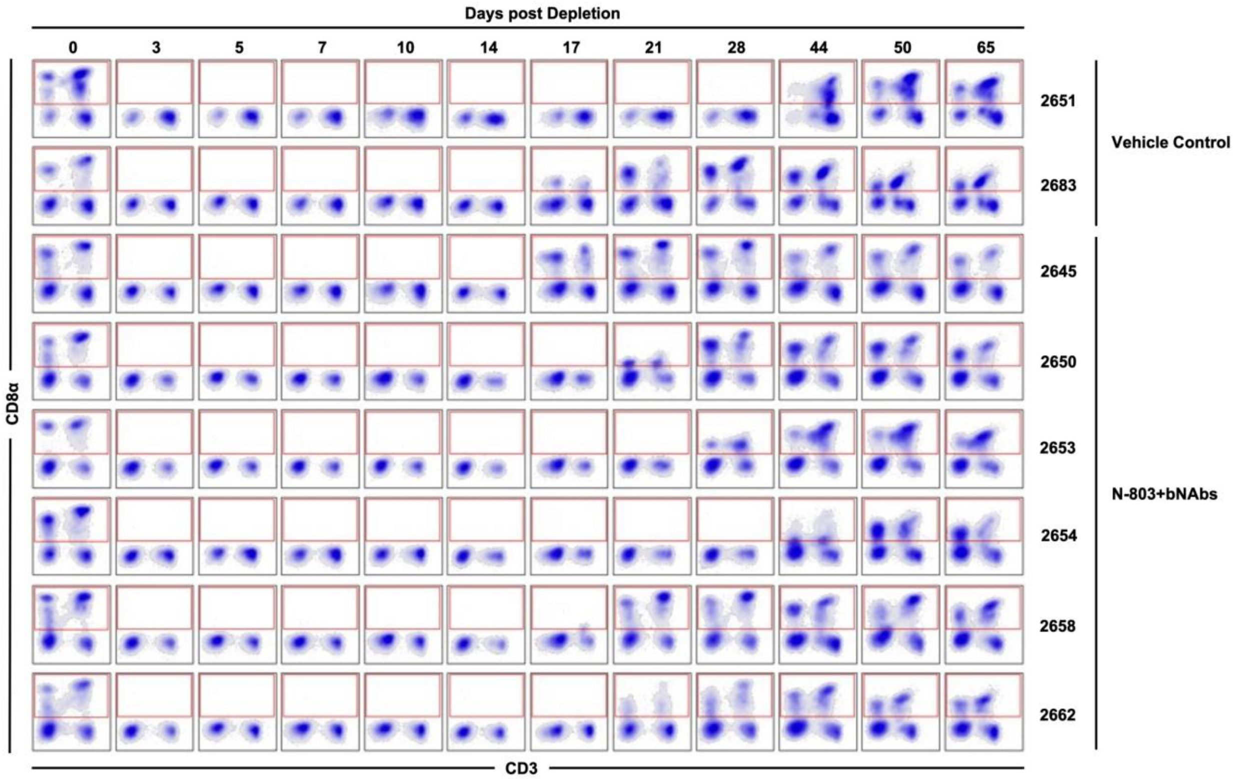

Fig. S9. In vivo CD8α⁺ lymphocyte depletion. CD8⁺ lymphocytes were depleted in vivo using the MT807R1 monoclonal antibody in two control RMs and six aviremic RMs treated with N-803+10-1074+3BNC117. The presence of CD8⁺ T cells in the peripheral blood was monitored by flow cytometry.

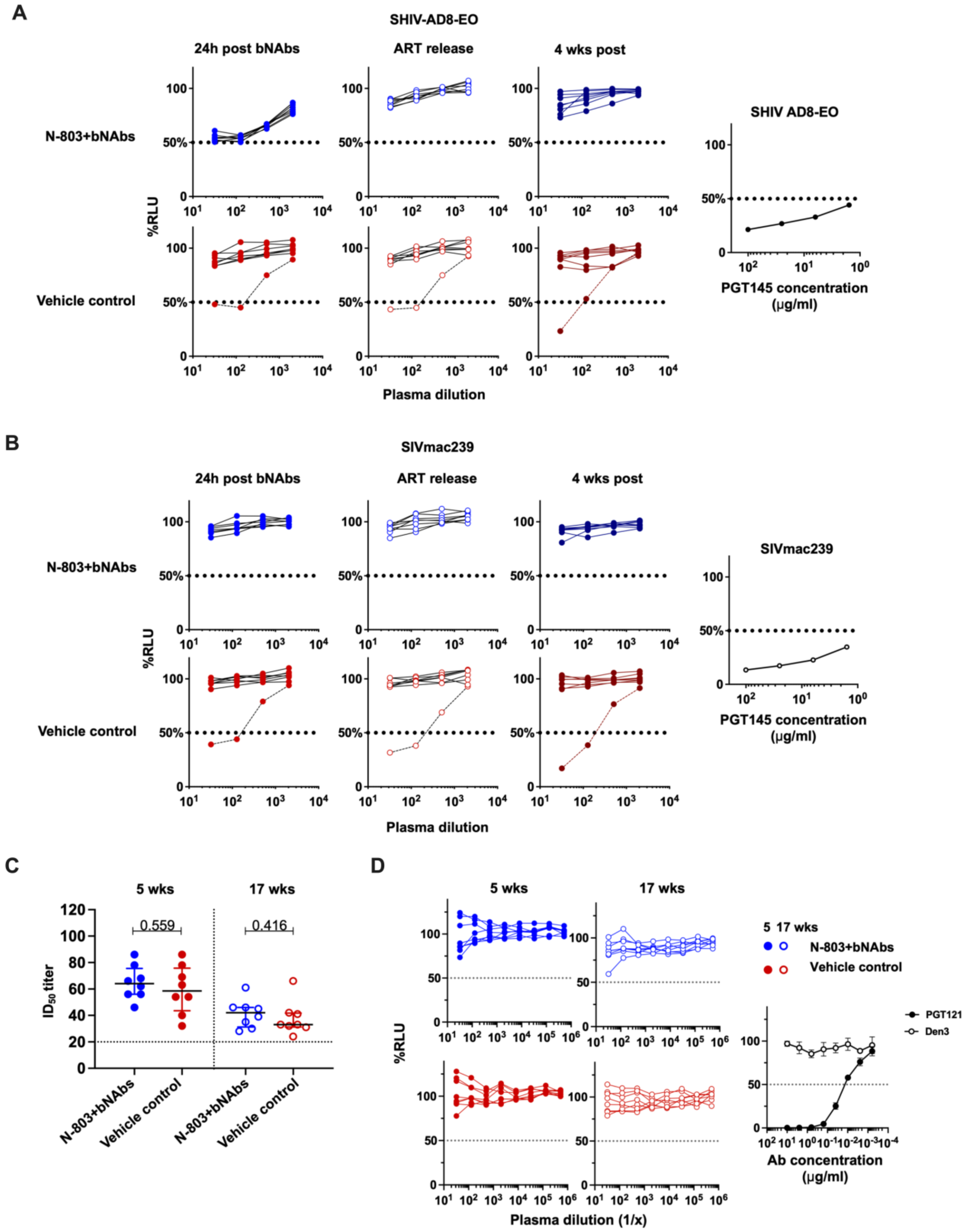

Fig. S10. Antibody-dependent cell-mediated cytotoxicity (ADCC) activity. In Study 2, (**A**) CEM.NKR-CCR5-sLTR-Luc cells were infected with SHIVAD8 (**B**) or SIVmac239 and incubated with an NK cell line expressing human CD16 at a 10:1 effector-to-target ratio with plasma isolated from RMs at three timepoints: 24 hours after the third bNAb dose, at the time of

ART discontinuation, and then 4 weeks after ART discontinuation. Plasma from animals administered N-803+10-1074+3BNC117 or formulation vehicle. ADCC activity was measured in relative light units (RLUs) in comparison to controls. Individual data points are means of triplicates. The dotted line indicates half-maximal lysis of infected cells. (**C**) Virus neutralization was measured using a luciferase-based assay in TZM.bl cells as ID_{50} titer or (**D**) as %RLU (using serial dilutions of plasma isolated at 5 and 17 weeks after ART discontinuation.

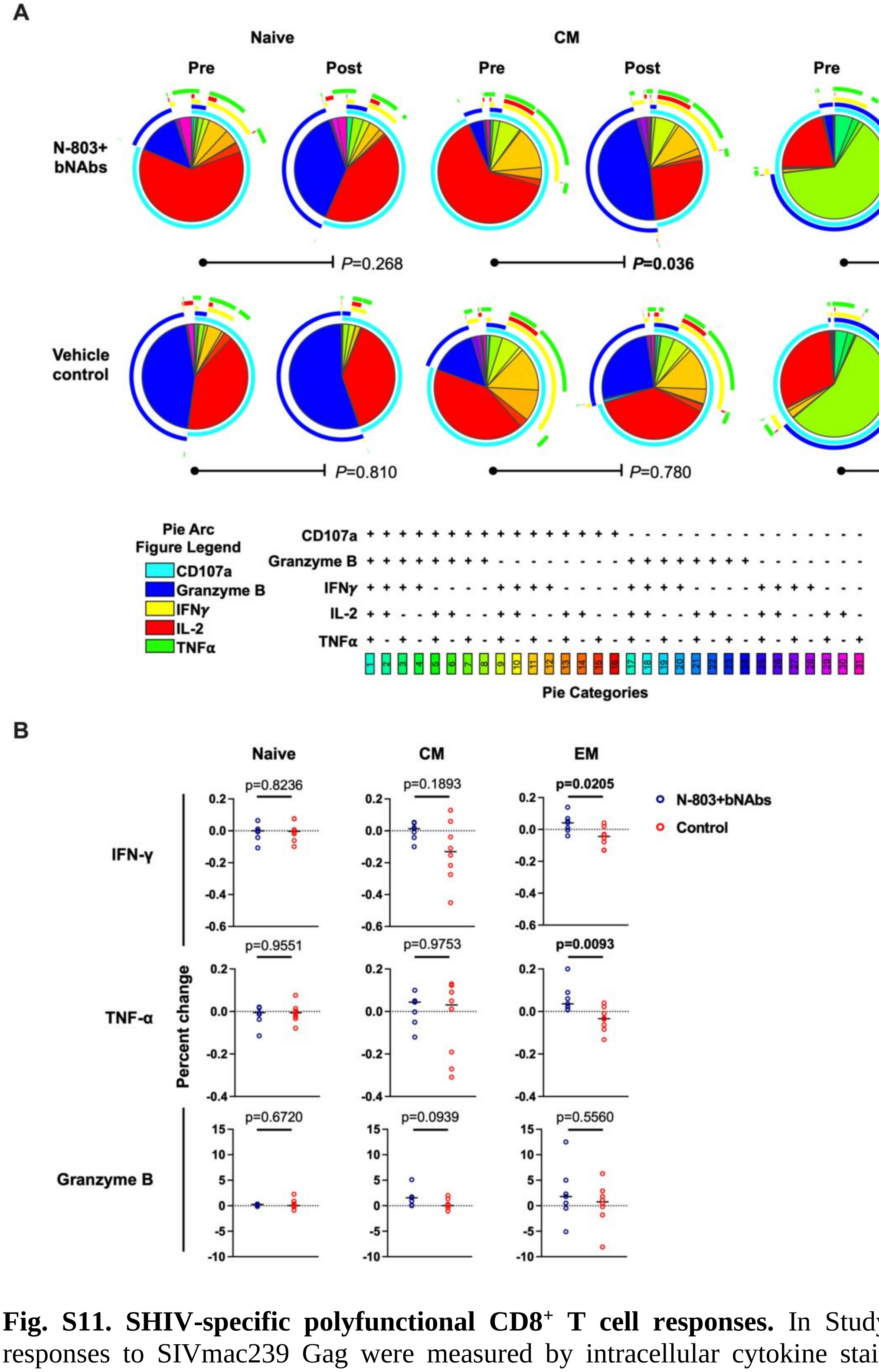

Fig. S11. SHIV-specific polyfunctional CD8$^+$ T cell responses. In Study 2, CD8$^+$ T cell responses to SIVmac239 Gag were measured by intracellular cytokine staining for CD107a, granzyme B, IFNγ, IL-2, and TNFα. (**A**) The pie charts show the mean frequency of cells performing all different combinations of effector function. The phenotypes of CD8$^+$ T cell subsets were determined: the fractions that fall into a naïve (CD95$^-$CD28$^+$), central memory (CM, CD95$^+$CD28$^+$), or effector memory (EM, CD95$^+$CD28$^-$) subset a shown in the pie charts. *P*-values were computed using a permutation test described in supplemental methods. (**B**) Values are

presented as the difference in percent change between pre-treatment and post-treatment in each group. Comparison between groups was determined using a Mann–Whitney U test.

A

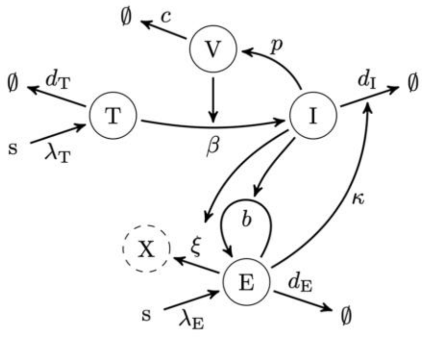
∅
c
V
p
∅
d_T
T
I
d_I
∅
s
λ_T
β
b
κ
X
ξ
E
d_E
s
λ_E
∅

B

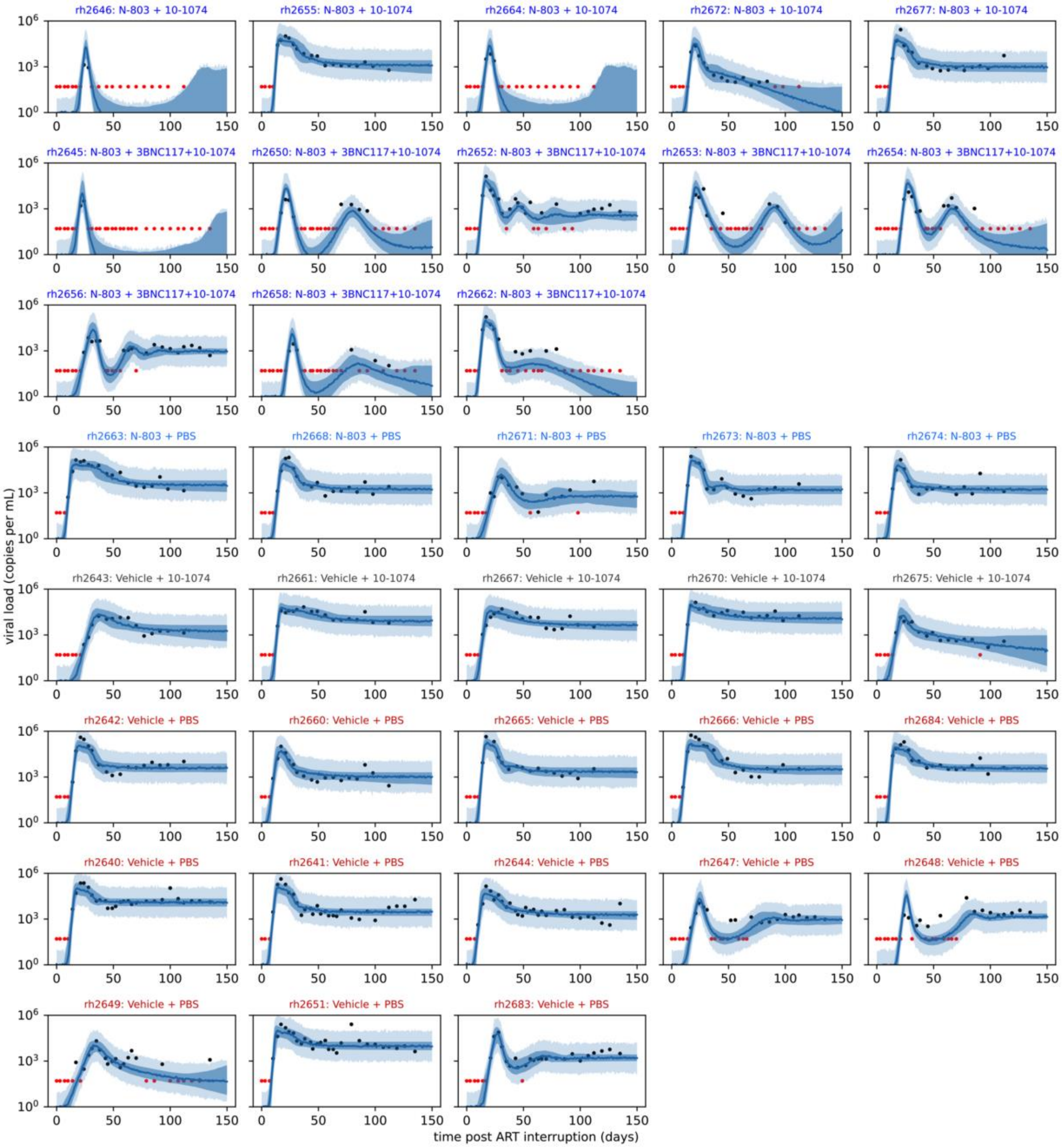
rh2646: N-803 + 10-1074
rh2655: N-803 + 10-1074
rh2664: N-803 + 10-1074
rh2672: N-803 + 10-1074
rh2677: N-803 + 10-1074
rh2645: N-803 + 3BNC117+10-1074
rh2650: N-803 + 3BNC117+10-1074
rh2652: N-803 + 3BNC117+10-1074
rh2653: N-803 + 3BNC117+10-1074
rh2654: N-803 + 3BNC117+10-1074
rh2656: N-803 + 3BNC117+10-1074
rh2658: N-803 + 3BNC117+10-1074
rh2662: N-803 + 3BNC117+10-1074
rh2663: N-803 + PBS
rh2668: N-803 + PBS
rh2671: N-803 + PBS
rh2673: N-803 + PBS
rh2674: N-803 + PBS
rh2643: Vehicle + 10-1074
rh2661: Vehicle + 10-1074
rh2667: Vehicle + 10-1074
rh2670: Vehicle + 10-1074
rh2675: Vehicle + 10-1074
rh2642: Vehicle + PBS
rh2660: Vehicle + PBS
rh2665: Vehicle + PBS
rh2666: Vehicle + PBS
rh2684: Vehicle + PBS
rh2640: Vehicle + PBS
rh2641: Vehicle + PBS
rh2644: Vehicle + PBS
rh2647: Vehicle + PBS
rh2648: Vehicle + PBS
rh2649: Vehicle + PBS
rh2651: Vehicle + PBS
rh2683: Vehicle + PBS
10^6
10^3
10^0
0
50
100
150
viral load (copies per mL)
time post ART interruption (days)

Fig. S12. Representative model fits and a diagram of the mathematical model of treatment induced viral suppression. (**A**) Diagram of the mathematical model. Target cells (T) are infected at a rate proportional to the number of infected cells (I). Infected cells are neutralized by effector cells (E), and they stimulate proliferation of effector cells. At high concentrations of infected cells, effector cells adopt an exhausted phenotype (X), which is not explicitly modeled. Additional parameters include target cell supply (λ_T) and death (d_T) rate; infected cell death (d_I) and elimination by effector cells (κE); effector cell entrance (λ_E), death (d_E), maximum proliferation (b), and maximum exhaustion (ξ) rate; virion production (p) and clearance (c); β, the infection rate constant; κ, the elimination rate constant. (**B**) Viral load trajectories of the fitted dynamical model. Shown are the viral load (VL) measurements following treatment interruption for each animal (black and red dots) and the fitted model predictions (blue curves and envelopes). The red dots indicate that the VL was below the limit of detection. To generate individual model trajectories and indicate their uncertainty for a given animal, we sample individual parameter values from the random effects distribution, conditional on the observed VL measurements. Using these samples, we generate an ensemble of likely trajectories. The blue curve is the median of this ensemble, and the dark blue envelope indicates the 2.5-to-97.5 percentiles. Using the ensemble of trajectories, we simulate VL measurements using a log-normal measurement error model. The 2.5-to-97.5 percentiles of these simulations are indicated by the light-blue envelopes.

Table S1. 10-1074 plasma concentrations (μg/ml) at each dose.

	Study 1			
**Group**	**10-1074**	**N-803+10-1074**	**10-1074**	**N-803+10-1074**
24 hours post	first dose	first dose	second dose	second dose
Minimum	110	93.7	170.4	35.3
**Median**	**134.6**	**164.8**	**220.5**	**181.9**
Maximum	194.1	181.8	495.7	230.5
**Mean**	**149.2**	**149.1**	**265.1**	**150.3**
SD	34.97	36.96	134.8	79.85

Table S2. 10-1074 and 3BNC117 plasma concentrations (µg/ml) at each dose.

	**Study 2**					
**bNAb**	**3BNC117**			**10-1074**		
24 hours post	first dose	second dose	third dose	first dose	second dose	third dose
Minimum	15.8	23.4	21.2	56.8	110.9	83
**Median**	**25.1**	**35.4**	**34**	**121**	**132**	**244.2**
Maximum	27.2	48.5	46	205.4	375.6	389.2
**Mean**	**22.26**	**35.48**	**33.79**	**121.6**	**178**	**242.5**
SD	5.251	8.63	8.093	42.46	92.59	99.29

Table S3. List of flow cytometry antibodies used for immune analysis.

Antibody	Clone	Fluorochrome	Manufacturer	Dilution
Anti-Human CD3	SP34-2	AF700	BD Bioscience	1:20
Anti-Human CD3	SP34-2	BUV395	BD Bioscience	1:20
Anti-Human CD3	SP34-2	V450	BD Bioscience	1:20
Anti-Human CD4	L200	BV510	BD Bioscience	1:100
Anti-Human CD4	L200	BV711	BD Bioscience	1:100
Anti-Human CD8	SK1	APC-Cy7	BD Bioscience	1:50
Anti-Human CD8	SK1	APC-H7	BD Bioscience	1:50
Anti-Human CD16	3G8	BV510	BD Bioscience	1:50
Anti-Human CD20	2H7	BUV395	BD Bioscience	1:50
Anti-Human CD20	2H7	BV570	Biolegend	1:50
Anti-Human CD20	2H7	BV650	BD Bioscience	1:100
Anti-Human CD25	M-A251	PE-Cy7	BD Bioscience	1:20
Anti-Human CD28	CD28.2	PE-CF594	BD Bioscience	1:20
Anti-Human CD28	CD28.2	PE-Cy7	BD Bioscience	1:20
Anti-Human CD28	CD28.2	PerCP-Cy5.5	BD Bioscience	1:50
Anti-NHP CD45	D058-1283	BUV737	BD Bioscience	1:50
Anti-NHP CD45	D058-1283	BV786	BD Bioscience	1:100
Anti-Human CD56	NCAM16.2	BV650	BD Bioscience	1:20
Anti-Human CD69	TP1.55.3	ECD	Beckman Coulter	1:20
Anti-Human CD95	DX2	BV711	BD Bioscience	1:50
Anti-Human CD95	DX2	PE	BD Bioscience	1:50
Anti-Human CD107a	H4A3	BV786	BD Bioscience	1:20
Anti-Human CD183 (CXCR3)	1C6/CXCR3	APC	BD Bioscience	1:50
Anti-Human CD185 (CXCR5)	MU5UBEE	PE-Cy7	eBioscience	1:20
Anti-Human CD197 (CCR7)	150503	FITC	BD Bioscience	1:20
Anti-Human CD278 (ICOS)	C398.4A	PerCP-Cy5.5	Biolegend	1:200
Anti-Human CD279 (PD-1)	EH12.2H7	BV421	Biolegend	1:50
Anti-Human HLA-DR	G46-6	APC-H7	BD Bioscience	1:80
Anti-Human NKG2A	Z199	PE-Cy7	Beckman Coulter	1:20
Anti-Human NKG2D	BAT221	PE	Miltenyi Biotec	1:50
Anti-Human NKp30	REA823	APC	Miltenyi Biotec	1:50
Anti-Human NKp46	BAB281	PerCP-Cy5.5	Beckman Coulter	1:50
Anti-Human Granzyme B	GB-11	AF700	BD Bioscience	1:50
Anti-Human IFN-γ	B27	BUV395	BD Bioscience	1:20
Anti-Human IL-2	MQ1-17H12	APC	BD Bioscience	1:5
Anti-Human TNF-α	Mab11	BV650	BD Bioscience	1:20
LIVE/DEAD Fixable Aqua			Invitrogen	1:1000

References and Notes

1. D. Finzi, J. Blankson, J. D. Siliciano, J. B. Margolick, K. Chadwick, T. Pierson, K. Smith, J. Lisziewicz, F. Lori, C. Flexner, T. C. Quinn, R. E. Chaisson, E. Rosenberg, B. Walker, S. Gange, J. Gallant, R. F. Siliciano, Latent infection of CD4+ T cells provides a mechanism for lifelong persistence of HIV-1, even in patients on effective combination therapy. *Nat. Med.* **5**, 512–517 (1999). doi:10.1038/8394 Medline

2. L. Zhang, B. Ramratnam, K. Tenner-Racz, Y. He, M. Vesanen, S. Lewin, A. Talal, P. Racz, A. S. Perelson, B. T. Korber, M. Markowitz, D. D. Ho, Quantifying residual HIV-1 replication in patients receiving combination antiretroviral therapy. *N. Engl. J. Med.* **340**, 1605–1613 (1999). doi:10.1056/NEJM199905273402101 Medline

3. T. W. Chun, R. T. Davey Jr., D. Engel, H. C. Lane, A. S. Fauci, Re-emergence of HIV after stopping therapy. *Nature* **401**, 874–875 (1999). doi:10.1038/44755 Medline

4. T. W. Chun, L. Carruth, D. Finzi, X. Shen, J. A. DiGiuseppe, H. Taylor, M. Hermankova, K. Chadwick, J. Margolick, T. C. Quinn, Y.-H. Kuo, R. Brookmeyer, M. A. Zeiger, P. Barditch-Crovo, R. F. Siliciano, Quantification of latent tissue reservoirs and total body viral load in HIV-1 infection. *Nature* **387**, 183–188 (1997). doi:10.1038/387183a0 Medline

5. T. W. Chun, L. Stuyver, S. B. Mizell, L. A. Ehler, J. A. M. Mican, M. Baseler, A. L. Lloyd, M. A. Nowak, A. S. Fauci, Presence of an inducible HIV-1 latent reservoir during highly active antiretroviral therapy. *Proc. Natl. Acad. Sci. U.S.A.* **94**, 13193–13197 (1997). doi:10.1073/pnas.94.24.13193 Medline

6. A. Sáez-Cirión, C. Bacchus, L. Hocqueloux, V. Avettand-Fenoel, I. Girault, C. Lecuroux, V. Potard, P. Versmisse, A. Melard, T. Prazuck, B. Descours, J. Guergnon, J.-P. Viard, F. Boufassa, O. Lambotte, C. Goujard, L. Meyer, D. Costagliola, A. Venet, G. Pancino, B. Autran, C. Rouzioux; ANRS VISCONTI Study Group, Post-treatment HIV-1 controllers with a long-term virological remission after the interruption of early initiated antiretroviral therapy ANRS VISCONTI Study. *PLOS Pathog.* **9**, e1003211 (2013). doi:10.1371/journal.ppat.1003211 Medline

7. K. Chamie, S. S. Chang, E. Kramolowsky, M. L. Gonzalgo, P. K. Agarwal, J. C. Bassett, M. Bjurlin, M. L. Cher, W. Clark, B. E. Cowan, R. David, E. Goldfischer, K. Guru, M. W. Jalkut, S. D. Kaffenberger, J. Kaminetsky, A. E. Katz, A. S. Koo, W. J. Sexton, S. N. Tikhonenkov, E. J. Trabulsi, A. F. Trainer, P. Spilman, M. Huang, P. Bhar, S. A. Taha, L. Sender, S. Reddy, P. Soon-Shiong, IL-15 superagonist NAI in BCG-unresponsive non–muscle-invasive bladder cancer. *NEJM Evid.* **2**, a2200167 (2023). Medline

8. J. B. McBrien, M. Mavigner, L. Franchitti, S. A. Smith, E. White, G. K. Tharp, H. Walum, K. Busman-Sahay, C. R. Aguilera-Sandoval, W. O. Thayer, R. A. Spagnuolo, M. Kovarova, A. Wahl, B. Cervasi, D. M. Margolis, T. H. Vanderford, D. G. Carnathan, M. Paiardini, J. D. Lifson, J. H. Lee, J. T. Safrit, S. E. Bosinger, J. D. Estes, C. A. Derdeyn, J. V. Garcia, D. A. Kulpa, A. Chahroudi, G. Silvestri, Robust and persistent reactivation of SIV and HIV by N-803 and depletion of $CD8^+$ cells. *Nature* **578**, 154–159 (2020). doi:10.1038/s41586-020-1946-0 Medline

9. J. S. Miller, Z. B. Davis, E. Helgeson, C. Reilly, A. Thorkelson, J. Anderson, N. S. Lima, S. Jorstad, G. T. Hart, J. H. Lee, J. T. Safrit, H. Wong, S. Cooley, L. Gharu, H. Chung, P. Soon-Shiong, C. Dobrowolski, C. V. Fletcher, J. Karn, D. C. Douek, T. W. Schacker, Safety and virologic impact of the IL-15 superagonist N-803 in people living with HIV: A phase 1 trial. *Nat. Med.* **28**, 392–400 (2022). doi:10.1038/s41591-021-01651-9 Medline

10. R. Romee, S. Cooley, M. M. Berrien-Elliott, P. Westervelt, M. R. Verneris, J. E. Wagner, D. J. Weisdorf, B. R. Blazar, C. Ustun, T. E. DeFor, S. Vivek, L. Peck, J. F. DiPersio, A. F. Cashen, R. Kyllo, A. Musiek, A. Schaffer, M. J. Anadkat, I. Rosman, D. Miller, J. O. Egan, E. K. Jeng, A. Rock, H. C. Wong, T. A. Fehniger, J. S. Miller, First-in-human phase 1 clinical study of the IL-15 superagonist complex ALT-803 to treat relapse after transplantation. *Blood* **131**, 2515–2527 (2018). doi:10.1182/blood-2017-12-823757 Medline

11. Y. Nishimura, R. Gautam, T.-W. Chun, R. Sadjadpour, K. E. Foulds, M. Shingai, F. Klein, A. Gazumyan, J. Golijanin, M. Donaldson, O. K. Donau, R. J. Plishka, A. Buckler-White, M. S. Seaman, J. D. Lifson, R. A. Koup, A. S. Fauci, M. C. Nussenzweig, M. A. Martin, Early antibody therapy can induce long-lasting immunity to SHIV. *Nature* **543**, 559–563 (2017). doi:10.1038/nature21435 Medline

12. J. F. Scheid, J. A. Horwitz, Y. Bar-On, E. F. Kreider, C.-L. Lu, J. C. C. Lorenzi, A. Feldmann, M. Braunschweig, L. Nogueira, T. Oliveira, I. Shimeliovich, R. Patel, L. Burke, Y. Z. Cohen, S. Hadrigan, A. Settler, M. Witmer-Pack, A. P. West Jr., B. Juelg, T. Keler, T. Hawthorne, B. Zingman, R. M. Gulick, N. Pfeifer, G. H. Learn, M. S. Seaman, P. J. Bjorkman, F. Klein, S. J. Schlesinger, B. D. Walker, B. H. Hahn, M. C. Nussenzweig, M. Caskey, HIV-1 antibody 3BNC117 suppresses viral rebound in humans during treatment interruption. *Nature* **535**, 556–560 (2016). doi:10.1038/nature18929 Medline

13. Y. Nishimura, O. K. Donau, J. Dias, S. Ferrando-Martinez, E. Jesteadt, R. Sadjadpour, R. Gautam, A. Buckler-White, R. Geleziunas, R. A. Koup, M. C. Nussenzweig, M. A. Martin, Immunotherapy during the acute SHIV infection of macaques confers long-term suppression of viremia. *J. Exp. Med.* **218**, e20201214 (2021). doi:10.1084/jem.20201214 Medline

14. J. D. Gunst, M. H. Pahus, M. Rosás-Umbert, I.-N. Lu, T. Benfield, H. Nielsen, I. S. Johansen, R. Mohey, L. Østergaard, V. Klastrup, M. Khan, M. H. Schleimann, R. Olesen, H. Støvring, P. W. Denton, N. N. Kinloch, D. C. Copertino, A. R. Ward, W. D. C. Alberto, S. D. Nielsen, M. C. Puertas, V. Ramos, J. D. Reeves, C. J. Petropoulos, J. Martinez-Picado, Z. L. Brumme, R. B. Jones, J. Fox, M. Tolstrup, M. C. Nussenzweig, M. Caskey, S. Fidler, O. S. Søgaard, Early intervention with 3BNC117 and romidepsin at antiretroviral treatment initiation in people with HIV-1: A phase 1b/2a, randomized trial. *Nat. Med.* **28**, 2424–2435 (2022). doi:10.1038/s41591-022-02023-7 Medline

15. M. C. Sneller, J. Blazkova, J. S. Justement, V. Shi, B. D. Kennedy, K. Gittens, J. Tolstenko, G. McCormack, E. J. Whitehead, R. F. Schneck, M. A. Proschan, E. Benko, C. Kovacs, C. Oguz, M. S. Seaman, M. Caskey, M. C. Nussenzweig, A. S. Fauci, S. Moir, T.-W. Chun, Combination anti-HIV antibodies provide sustained virological suppression. *Nature* **606**, 375–381 (2022). doi:10.1038/s41586-022-04797-9 Medline

16. J. Niessl, A. E. Baxter, P. Mendoza, M. Jankovic, Y. Z. Cohen, A. L. Butler, C.-L. Lu, M. Dubé, I. Shimeliovich, H. Gruell, F. Klein, M. Caskey, M. C. Nussenzweig, D. E. Kaufmann, Combination anti-HIV-1 antibody therapy is associated with increased virus-specific T cell immunity. *Nat. Med.* **26**, 222–227 (2020). doi:10.1038/s41591-019-0747-1 Medline

17. M. Rosás-Umbert, J. D. Gunst, M. H. Pahus, R. Olesen, M. Schleimann, P. W. Denton, V. Ramos, A. Ward, N. N. Kinloch, D. C. Copertino, T. Escribà, A. Llano, Z. L. Brumme, R. Brad Jones, B. Mothe, C. Brander, J. Fox, M. C. Nussenzweig, S. Fidler, M. Caskey, M. Tolstrup, O. S. Søgaard, Administration of broadly neutralizing anti-HIV-1 antibodies at ART initiation maintains long-term CD8$^+$ T cell immunity. *Nat. Commun.* **13**, 6473 (2022). doi:10.1038/s41467-022-34171-2 Medline

18. C. Gaebler, L. Nogueira, E. Stoffel, T. Y. Oliveira, G. Breton, K. G. Millard, M. Turroja, A. Butler, V. Ramos, M. S. Seaman, J. D. Reeves, C. J. Petroupoulos, I. Shimeliovich, A. Gazumyan, C. S. Jiang, N. Jilg, J. F. Scheid, R. Gandhi, B. D. Walker, M. C. Sneller, A. Fauci, T.-W. Chun, M. Caskey, M. C. Nussenzweig, Prolonged viral suppression with anti-HIV-1 antibody therapy. *Nature* **606**, 368–374 (2022). doi:10.1038/s41586-022-04597-1 Medline

19. J. B. Whitney, A. L. Hill, S. Sanisetty, P. Penaloza-MacMaster, J. Liu, M. Shetty, L. Parenteau, C. Cabral, J. Shields, S. Blackmore, J. Y. Smith, A. L. Brinkman, L. E. Peter, S. I. Mathew, K. M. Smith, E. N. Borducchi, D. I. S. Rosenbloom, M. G. Lewis, J. Hattersley, B. Li, J. Hesselgesser, R. Geleziunas, M. L. Robb, J. H. Kim, N. L. Michael, D. H. Barouch, Rapid seeding of the viral reservoir prior to SIV viraemia in rhesus monkeys. *Nature* **512**, 74–77 (2014). doi:10.1038/nature13594 Medline

20. A. Sáez-Cirión, S. Y. Shin, P. Versmisse, F. Barré-Sinoussi, G. Pancino, Ex vivo T cell-based HIV suppression assay to evaluate HIV-specific CD8+ T-cell responses. *Nat. Protoc.* **5**, 1033–1041 (2010). doi:10.1038/nprot.2010.73 Medline

21. T. Schoofs, F. Klein, M. Braunschweig, E. F. Kreider, A. Feldmann, L. Nogueira, T. Oliveira, J. C. C. Lorenzi, E. H. Parrish, G. H. Learn, A. P. West Jr., P. J. Bjorkman, S. J. Schlesinger, M. S. Seaman, J. Czartoski, M. J. McElrath, N. Pfeifer, B. H. Hahn, M. Caskey, M. C. Nussenzweig, HIV-1 therapy with monoclonal antibody 3BNC117 elicits host immune responses against HIV-1. *Science* **352**, 997–1001 (2016). doi:10.1126/science.aaf0972 Medline

22. M. Caskey, F. Klein, M. C. Nussenzweig, Broadly neutralizing anti-HIV-1 monoclonal antibodies in the clinic. *Nat. Med.* **25**, 547–553 (2019). doi:10.1038/s41591-019-0412-8 Medline

23. J. Blazkova, F. Gao, M. H. Marichannegowda, J. S. Justement, V. Shi, E. J. Whitehead, R. F. Schneck, E. D. Huiting, K. Gittens, M. Cottrell, E. Benko, C. Kovacs, J. Lack, M. C. Sneller, S. Moir, A. S. Fauci, T.-W. Chun, Distinct mechanisms of long-term virologic control in two HIV-infected individuals after treatment interruption of anti-retroviral therapy. *Nat. Med.* **27**, 1893–1898 (2021). doi:10.1038/s41591-021-01503-6 Medline

24. F. Lori, M. G. Lewis, J. Xu, G. Varga, D. E. Zinn Jr., C. Crabbs, W. Wagner, J. Greenhouse, P. Silvera, J. Yalley-Ogunro, C. Tinelli, J. Lisziewicz, Control of SIV rebound through

structured treatment interruptions during early infection. *Science* **290**, 1591–1593 (2000). doi:10.1126/science.290.5496.1591 Medline

25. H. J. Barbian, M. S. Seaton, S. D. Narasipura, J. Wallace, R. Rajan, B. E. Sha, L. Al-Harthi, β-catenin regulates HIV latency and modulates HIV reactivation. *PLOS Pathog.* **18**, e1010354 (2022). doi:10.1371/journal.ppat.1010354 Medline

26. J. Wallace, S. D. Narasipura, B. E. Sha, A. L. French, L. Al-Harthi, Canonical Wnts mediate $CD8^+$ T cell noncytolytic anti-HIV-1 activity and correlate with HIV-1 clinical status. *J. Immunol.* **205**, 2046–2055 (2020). doi:10.4049/jimmunol.1801379 Medline

27. M. Zanoni, D. Palesch, C. Pinacchio, M. Statzu, G. K. Tharp, M. Paiardini, A. Chahroudi, S. E. Bosinger, J. Yoon, B. Cox, G. Silvestri, D. A. Kulpa, Innate, non-cytolytic CD8+ T cell-mediated suppression of HIV replication by MHC-independent inhibition of virus transcription. *PLOS Pathog.* **16**, e1008821 (2020). doi:10.1371/journal.ppat.1008821 Medline

28. Y. Nishimura, M. Shingai, R. Willey, R. Sadjadpour, W. R. Lee, C. R. Brown, J. M. Brenchley, A. Buckler-White, R. Petros, M. Eckhaus, V. Hoffman, T. Igarashi, M. A. Martin, Generation of the pathogenic R5-tropic simian/human immunodeficiency virus SHIVAD8 by serial passaging in rhesus macaques. *J. Virol.* **84**, 4769–4781 (2010). doi:10.1128/JVI.02279-09 Medline

29. L. J. Yant, T. C. Friedrich, R. C. Johnson, G. E. May, N. J. Maness, A. M. Enz, J. D. Lifson, D. H. O'Connor, M. Carrington, D. I. Watkins, The high-frequency major histocompatibility complex class I allele Mamu-B*17 is associated with control of simian immunodeficiency virus SIVmac239 replication. *J. Virol.* **80**, 5074–5077 (2006). doi:10.1128/JVI.80.10.5074-5077.2006 Medline

30. B. R. Mothé, J. Weinfurter, C. Wang, W. Rehrauer, N. Wilson, T. M. Allen, D. B. Allison, D. I. Watkins, Expression of the major histocompatibility complex class I molecule Mamu-A*01 is associated with control of simian immunodeficiency virus SIVmac239 replication. *J. Virol.* **77**, 2736–2740 (2003). doi:10.1128/JVI.77.4.2736-2740.2003 Medline

31. J. T. Loffredo, J. Maxwell, Y. Qi, C. E. Glidden, G. J. Borchardt, T. Soma, A. T. Bean, D. R. Beal, N. A. Wilson, W. M. Rehrauer, J. D. Lifson, M. Carrington, D. I. Watkins, Mamu-B*08-positive macaques control simian immunodeficiency virus replication. *J. Virol.* **81**, 8827–8832 (2007). doi:10.1128/JVI.00895-07 Medline

32. D. C. Montefiori, Evaluating neutralizing antibodies against HIV, SIV, and SHIV in luciferase reporter gene assays. *Curr. Protoc. Immunol.* **Chapter 12**, 12.11.1–12.11.17 (2005). doi:10.1002/0471142735.im1211s64 Medline

33. J. B. Whitney, S.-Y. Lim, C. E. Osuna, J. L. Kublin, E. Chen, G. Yoon, P.-T. Liu, P. Abbink, E. N. Borducci, A. Hill, M. G. Lewis, R. Geleziunas, M. L. Robb, N. L. Michael, D. H. Barouch, Prevention of SIVmac251 reservoir seeding in rhesus monkeys by early antiretroviral therapy. *Nat. Commun.* **9**, 5429 (2018). doi:10.1038/s41467-018-07881-9 Medline

34. C. J. Pitcher, S. I. Hagen, J. M. Walker, R. Lum, B. L. Mitchell, V. C. Maino, M. K. Axthelm, L. J. Picker, Development and homeostasis of T cell memory in rhesus macaque. *J. Immunol.* **168**, 29–43 (2002). doi:10.4049/jimmunol.168.1.29 Medline

35. M. Sarzotti-Kelsoe, R. T. Bailer, E. Turk, C. L. Lin, M. Bilska, K. M. Greene, H. Gao, C. A. Todd, D. A. Ozaki, M. S. Seaman, J. R. Mascola, D. C. Montefiori, Optimization and validation of the TZM-bl assay for standardized assessments of neutralizing antibodies against HIV-1. *J. Immunol. Methods* **409**, 131–146 (2014). doi:10.1016/j.jim.2013.11.022 Medline

36. Y. Nishimura, R. Sadjadpour, J. J. Mattapallil, T. Igarashi, W. Lee, A. Buckler-White, M. Roederer, T.-W. Chun, M. A. Martin, High frequencies of resting CD4+ T cells containing integrated viral DNA are found in rhesus macaques during acute lentivirus infections. *Proc. Natl. Acad. Sci. U.S.A.* **106**, 8015–8020 (2009). doi:10.1073/pnas.0903022106 Medline

37. A. M. Bender, F. R. Simonetti, M. R. Kumar, E. J. Fray, K. M. Bruner, A. E. Timmons, K. Y. Tai, K. M. Jenike, A. A. R. Antar, P.-T. Liu, Y.-C. Ho, D. N. Raugi, M. Seydi, G. S. Gottlieb, A. A. Okoye, G. Q. Del Prete, L. J. Picker, J. L. Mankowski, J. D. Lifson, J. D. Siliciano, G. M. Laird, D. H. Barouch, J. E. Clements, R. F. Siliciano, The Landscape of Persistent Viral Genomes in ART-Treated SIV, SHIV, and HIV-2 Infections. *Cell Host Microbe* **26**, 73–85.e4 (2019). doi:10.1016/j.chom.2019.06.005 Medline

38. K. M. Bruner, Z. Wang, F. R. Simonetti, A. M. Bender, K. J. Kwon, S. Sengupta, E. J. Fray, S. A. Beg, A. A. R. Antar, K. M. Jenike, L. N. Bertagnolli, A. A. Capoferri, J. T. Kufera, A. Timmons, C. Nobles, J. Gregg, N. Wada, Y.-C. Ho, H. Zhang, J. B. Margolick, J. N. Blankson, S. G. Deeks, F. D. Bushman, J. D. Siliciano, G. M. Laird, R. F. Siliciano, A quantitative approach for measuring the reservoir of latent HIV-1 proviruses. *Nature* **566**, 120–125 (2019). doi:10.1038/s41586-019-0898-8 Medline

39. D. M. Margolis, Eradication therapies for HIV Infection: Time to begin again. *AIDS Res. Hum. Retroviruses* **27**, 347–353 (2011). doi:10.1089/aid.2011.0017 Medline

40. J. E. Schmitz, M. A. Simon, M. J. Kuroda, M. A. Lifton, M. W. Ollert, C.-W. Vogel, P. Racz, K. Tenner-Racz, B. J. Scallon, M. Dalesandro, J. Ghrayeb, E. P. Rieber, V. G. Sasseville, K. A. Reimann, A nonhuman primate model for the selective elimination of CD8+ lymphocytes using a mouse-human chimeric monoclonal antibody. *Am. J. Pathol.* **154**, 1923–1932 (1999). doi:10.1016/S0002-9440(10)65450-8 Medline

41. M. M. Donaldson, S.-F. Kao, L. Eslamizar, C. Gee, G. Koopman, M. Lifton, J. E. Schmitz, A. W. Sylwester, A. Wilson, N. Hawkins, S. G. Self, M. Roederer, K. E. Foulds, Optimization and qualification of an 8-color intracellular cytokine staining assay for quantifying T cell responses in rhesus macaques for pre-clinical vaccine studies. *J. Immunol. Methods* **386**, 10–21 (2012). doi:10.1016/j.jim.2012.08.011 Medline

42. M. D. Alpert, L. N. Heyer, D. E. J. Williams, J. D. Harvey, T. Greenough, M. Allhorn, D. T. Evans, A novel assay for antibody-dependent cell-mediated cytotoxicity against HIV-1- or SIV-infected cells reveals incomplete overlap with antibodies measured by neutralization and binding assays. *J. Virol.* **86**, 12039–12052 (2012). doi:10.1128/JVI.01650-12 Medline

43. M. S. Seaman, D. F. Leblanc, L. E. Grandpre, M. T. Bartman, D. C. Montefiori, N. L. Letvin, J. R. Mascola, Standardized assessment of NAb responses elicited in rhesus monkeys immunized with single- or multi-clade HIV-1 envelope immunogens. *Virology* **367**, 175–186 (2007). doi:10.1016/j.virol.2007.05.024 Medline

44. A. S. Perelson, A. U. Neumann, M. Markowitz, J. M. Leonard, D. D. Ho, HIV-1 dynamics in vivo: Virion clearance rate, infected cell life-span, and viral generation time. *Science* **271**, 1582–1586 (1996). doi:10.1126/science.271.5255.1582 Medline

45. J. M. Conway, A. S. Perelson, Post-treatment control of HIV infection. *Proc. Natl. Acad. Sci. U.S.A.* **112**, 5467–5472 (2015). doi:10.1073/pnas.1419162112 Medline

46. R. J. De Boer, A. S. Perelson, Towards a general function describing T cell proliferation. *J. Theor. Biol.* **175**, 567–576 (1995). doi:10.1006/jtbi.1995.0165 Medline

47. A. Mayer, Y. Zhang, A. S. Perelson, N. S. Wingreen, Regulation of T cell expansion by antigen presentation dynamics. *Proc. Natl. Acad. Sci. U.S.A.* **116**, 5914–5919 (2019). doi:10.1073/pnas.1812800116 Medline

48. R. W. Sanders, R. Derking, A. Cupo, J.-P. Julien, A. Yasmeen, N. de Val, H. J. Kim, C. Blattner, A. T. de la Peña, J. Korzun, M. Golabek, K. de Los Reyes, T. J. Ketas, M. J. van Gils, C. R. King, I. A. Wilson, A. B. Ward, P. J. Klasse, J. P. Moore, A next-generation cleaved, soluble HIV-1 Env trimer, BG505 SOSIP.664 gp140, expresses multiple epitopes for broadly neutralizing but not non-neutralizing antibodies. *PLOS Pathog.* **9**, e1003618 (2013). doi:10.1371/journal.ppat.1003618 Medline
